# Global pharmaceutical regulation: the challenge of integration for developing states

**DOI:** 10.1186/s12992-016-0208-2

**Published:** 2016-12-20

**Authors:** Anthony Pezzola, Cassandra M. Sweet

**Affiliations:** Instituto de Ciencia Política, Pontificia Universidad Católica de Chile, Av. Vicuña Mackenna 4860, Campus San Joaquín, Macul, 7820436 Santiago Chile

**Keywords:** Pharmaceuticals, Regulation, Access to medicine, Developing countries, World Health Organization

## Abstract

**Background:**

This paper has set out to map the state of pharmaceutical regulation in the developing world through the construction of cross-national indices drawing from World Health Organization data. The last two decades have been characterized by deep changes for the pharmaceutical sector, including the complete transformation of intellectual property systems at the behest of the World Trade Organization and the consolidation of global active ingredient suppliers in China and India. Although the rules for ownership of medicine have been set and globally implemented, we know surprisingly little about how the standards for market entrance and regulation of pharmaceutical products have changed at the national level. How standardized are national pharmaceutical market systems? Do we find homogeneity or variation across the developing world? Are their patterns for understanding why some countries have moved closer to one global norm for pharmaceutical regulation and others have developed hybrid models for oversight of this sector? Access to medicine is a core tool in public health. This paper gauges the levels of standards in public and private generics markets for developing countries building on national-level pharmaceutical market surveys for 78 countries to offer three indicators of market oversight: State Regulatory Infrastructure, Monitoring the Private Market and Public Quality Control. Identifying the different variables that affect a state’s institutional capacity and current standard level offers new insights to the state of pharmaceuticals in the developing world. It is notable that there are very few (none at the time of this paper) studies that map out the new global terrain for pharmaceutical regulation in the post-TRIPS context.

**Results:**

This paper uses item response theory to develop original indicators of pharmaceutical regulation. We find remarkable resistance to the implementation of global pharmaceutical norms for quality standards in developing states and in regulatory infrastructure. Human capacity across many developing countries remains limited. Most notably, variation among states is stark. Countries that have been leaders in establishing global norms do not appear to have influenced their neighbors in establishing regional patterns. Finally, in contrast to traditional theories of international norms diffusion, global standard-setters such as the United States or European Union appear to have surprisingly little influence on standard setting across our survey.

**Conclusions:**

Our research has implications for the framing of technical support on public health initiatives aimed at strengthening local public institutions in medicine and offers a new methodological approach for analyzing drug regulation systems in developing countries.

**Electronic supplementary material:**

The online version of this article (doi:10.1186/s12992-016-0208-2) contains supplementary material, which is available to authorized users.

## Background

The notion that health and access to medicine is central in human development has become axiomatic across development and international relations literatures. An abundance of recent work has focused on the question of access to medicine, nevertheless, scant research has looked at the state systems ensuring the quality standards for that medicine.^1^ This is particularly notable in terms of the relationship between globalization and the emergence of global norms for pharmaceutical quality. In the area of pharmaceutical regulation almost all cross-national empirical research has focused on intellectual property (IP) rights, leaving aside the question of the state’s capacity to regulate the pharmaceutical market and variations in regulatory practices across countries.^2^ It would be no understatement to note that since the implementation of the World Trade Organization’s Agreement (WTO) on Trade-Related Intellectual Property Rights (TRIPS), research on intellectual property and its effects on pharmaceutical markets has taken on a diluvial nature. Scholars have written about the process through which the Treaty was negotiated, [1–3], the resulting backlash at Doha [4, 5], the increasing “tightening” of the TRIPS regime [6, 7], the process of TRIPS implementation [8–11] and the flexibilities pursued under the treaty [12–14]. While these studies have made important contributions to our understanding of the global and national pharmaceutical markets, their focus on drug ownership rights has overlooked the capacity of states to regulate the quality of the drugs consumed by their citizens.

Evidence shows however that while countries have been transforming their norms on IP, they have also been undergoing profound regulatory restructuring of their pharmaceutical markets in terms of product registry and regulation. This aspect of pharmaceutical oversight, frequently referred to as pharmacovigelence, may prove to be the next frontier of global public goods negotiation for access to medicine [15].

This paper builds on a new stream of research in the global regulation and the access to medicine literatures by constructing three indices that improve our understanding of how pharmaceutical regulation is taking shape in the developing world. Our research question is simple: how does the quality of pharmaceutical regulation vary within and across countries? Using item response theory to construct indices of three dimensions of regulatory quality, this research offers the first cross-national measure of regulatory quality and the first estimate of variation within and across countries.

These indices are based on the World Health Organization (WHO) Country Pharmaceutical Situation 2011 survey of 78 small and developing countries (see Addition file 1 for a list of countries). The indices offer global reach, and key data on the evolving regulations in developing and least-developing countries. That said, the WHO survey and therefore, our results, do have limits: we do not provide a score of how well governments are in practice regulating these markets—the actual enforcement of the rules that they may have adopted. This research lends insight into the *de jure* variation within and across countries on three central dimensions relating to the control of quality in the production and distribution of medicine. A conceptual and empirical mapping of pharmaceutical regulatory standards in the developing world will help us make important strides toward understanding why some countries have been better able to regulate their markets and protect consumers. The setting of rules and norms is critical to protecting the interests of public health. At the same time, the standardization of good practices by governments offers civil society as well as manufactures, distributors, and retailers a pathway for ensuring transparent guidelines for monitoring and developing well-functioning public and private markets for medicines.

The paper proceeds as follows: the Background  section offers a brief review of the access to medicine literatures and the dynamics of global pharmaceutical regulation over recent decades, the Methods section describes the methods and data used to develop our indicies, the Results section introduces our indices and preliminary results. The Discussion section provides a brief discussion of these results, highlighting the variation in regulatory quality within and between countries, and sets out a pathway for future research on the causal mechanisms that might account for the patterns in global pharmaceutical regulation among small and developing countries as identified in this paper.

### Background: advances in theory and empirics of pharmaceutical regulation

The current global framework for pharmaceutical regulation has undergone a profound transformation over the past two decades. Implementation of the new global system for regulation of intellectual property standards since the mid-1990s has pushed developing states to establish new institutions to review, approve, and manage the emerging intellectual property rights system [2, 11, 16]. As a result, the pharmaceutical sector and its governance has become a flashpoint for conflict over the ownership rights, access, and marketing of pharmaceutical products [17].

Recent research has shown that as developing states have been pushed to implement IP standards, a concomitant wave of regulation of off-patent products has ensued [18]. Nevertheless, pharmaceutical standards and regulatory systems across the world remain in many countries fragile, uneven and highly dependent on aid and technical support from international donors. The weakness of the regulatory apparatus in many pharmaceutical markets was recently illustrated in a study that looked at least developed countries. Olsson et al. (2010) found that less than half of least-developed countries publically funded their pharmaceutical regulatory agencies [19]. Moreover, many state institutions with the mandate to approve, review, and regulate pharmaceutical products in the survey were found to be highly dependent on financing from international organizations. In one-third of the countries studied by Olsson et al., these activities were entirely financed by one international source: the Global Fund to fight AIDS, Tuberculosis and Malaria. Such studies lend weight to a growing scholarship on the developing world which suggests that quality regulation is inherently tied to a more pervasive lack of local, democratic control of health systems in developing countries [20]. Evidence that international institutions are central in supporting regulatory agencies speaks to the fragility and diversity of regulatory models across developing states.

Both the push to regulate ownership of pharmaceutical knowledge at a global level and the subsequent double movement of states to examine their regulatory structure at a local level opens new doors for theoretical and empirical research on regulation of standards for medicine. It is notable that there are very few (none at the time of this paper) studies that, in light of the seismic shifts in the pharmaceutical sector, map out the new global terrain for pharmaceutical regulation.^3^ We know very little about the diversity of regulatory systems and how they are enforced in the developing world. Which countries now regulate more rigorously the quality standards for medicines in their markets and why? Do patterns of adoption in emerging markets mimic those standards in developed markets? Why are some countries moving toward one global norm while others appear to build regulatory enclaves, rejecting global standards, and implementing national market requirements which are unique? What accounts for patterns of adherence and resistance to dominant pharmacological standards?

Although highly uneven levels of regulation persist across pharmaceutical markets, many scholars studying global regulatory states point to patterns of diffusion driven by advanced states [21, 22]. They suggest that key market players drive international norms. That industrialized countries seem to instate and then extend dominant regulatory standards fits both a commercial and political rationale. Firms operating across borders face lower transaction costs when, for example, the EU’s European Medicine’s Agency and the United State’s Food and Drug Administration share the same standards for bioequivalency and accept data from the same certified laboratories [23]. Consumers, be they individuals or public procurement entities, should in principle, benefit from the lower costs faced by these firms. A generic company base in Hyderabad, in theory, benefits when Brazil has standards equal to those in markets where the firm already operations because the barrier to market entrance is lowered. Though there appears to be a “new regulatory order” of independent regulatory authorities emerging in mid-level developed countries [24, 25], it is unclear that the particularly “sticky” pharmaceutical sector is adopting homogenous standards [10, 26].

At the center of this debate is a longstanding wave of research that has examined patterns of regulatory diffusion. Scholars have questioned why some countries adopt similar standards while others resist trends. The pharmaceutical market appears to have particularities that distinguish it fundamentally from other economies or products. The political economy of medicine is markedly different, as economist Daniel Carpenter has eloquently written, because neither the consumer, nor the regulatory agency can be entirely sure of the product’s quality. “The most salient institutional distinction rests in gatekeeping: the necessity of governmental pre-market review for new products, where any approval is based in part upon an experimental (non-market) history of the product” [27]. Moreover, regulators must guarantee the quality of approved drugs that reach consumers. This makes quality control an essential aspect of pharmaceutical regulation.

Gatekeeping of pharmaceutical markets has important political and public health implications. Brazil for example has been widely heralded for its successful program of ensuring universal access to antiretroviral medicine since the mid nineties [28, 29]. Yet, its public production and distribution has relied not only on constant negotiations with patent holding multinationals [30–32] but also with Indian and Chinese suppliers of active pharmaceutical ingredients in order to manufacture generic production. This negotiation, and a lack of quality standards for bulk pharmaceutical suppliers has been costly for the national program; for some public production facilities, the failure of the process has resulted in up to a third of all purchases being lost because of ineffectiveness [33].

The global rules now governing intellectual property have spurred a new set of challenges for developing states; these challenges are reinforced by the geographic distribution of pharmaceutical production and local institutional and political capacity. A ‘frenzy’ of mergers and acquisitions activities over the last two decades has resulted in unprecedented consolidation across all levels of the global pharmaceutical supply chain [34, 35]. Fewer suppliers have increased competitive pressure for the region’s public procurement programs and for local producers [36]. At the same time, so-called second-tier suppliers have flooded the international pharmaceutical market with products which may not meet quality standards. States face a double-edged regulatory challenge, harmonizing their intellectual property rights norms to global standards and working to upgrade and implement quality standards for local populations.

## Methods

Seeking to map out variation in pharmaceutical regulation within and across countries, this paper introduces three indices based on the WHO Country Pharmaceutical Situation 2011 survey.^4^ Survey questions directly related to three central dimensions related to the control and quality of the production and distribution of medicines have been identified in order to assess the ability of governments to ensure the quality of the medicines consumed by their citizens. The first index measures the existence of basic *State Regulatory Infrastructure*. The second index assesses the degree of statutory control over private transactions: *Monitoring the Private Market*. As previously discussed, the survey responses do not permit us to measure how well countries regulate and monitor their markets since they do not provide information about how well laws and policies are implemented. What these indices do allow us to measure is whether countries have adopted polices widely seen as desirable and positively associated with ensuring the quality and efficacy of medicines. The third index, *Public Quality Control*, evaluates the existence of polices associated with high standards of control of the public pharmaceutical market.

These three indices do not cover all aspects of pharmaceutical regulation and monitoring nor all practices necessary to ensure quality medicines. However, they include the most basic institutions, policies, and actions necessary to ensure the safety, efficacy, and quality of medicines. They also cover the key regulatory functions of licensing, inspection, product assessment, and registration as well as key policies associated with pharmacovigilance, all of which have been widely recognized as central to the protection of public health.^5^ Each of these functions relates to a different aspect of the market of medicines and each must be undertaken to ensure access to quality medications.

Basic regulatory authorities and policies are essential to the implementation and enforcement of any pharmaceutical policy in the public or private sector. The dimension *State Regulatory Infrastructure* takes into account the existence of a national medicines policy and of a medicines regulatory authority as well as the existence of established good practices in manufacturing, distribution, and retail. Especially in developing countries, ensuring access to quality medicines is complicated by complex and interdependent problems, which are best addressed using a common framework. A national medicines policy is a first step in identifying and resolving problems within the pharmaceutical sector. Medicine regulatory authorities (MRAs), whatever their official names, are a first step toward to ensuring a strong regulation of the market and the protection of public health; without a central authority monitoring, testing, and tracking medicines as well as licensing and inspecting pharmacists and points of distribution even the best statues can become ineffective. The publication of good practices by the government not only provides manufactures, distributors, and retailers a clear guide for action; it also provides regulators clear guidelines for monitoring the market and for implementing policies that will help develop well-functioning market for medicines.^6^

The index *State Regulatory Infrastructure* uses fourteen indicators from the WHO survey, which are detailed in Table 1.

**Table 1:** Composition of *State Regulatory Infrastructure*

3.01.04	Equal to one if a national medicines policy official document exists.
3.01.12	Equal to one if pharmaceutical policy implementation is being regularly monitored/assessed.
3.01.14	Equal to one if a policy is in place to manage and sanction conflict of interest issues in pharmaceutical affairs.
3.01.16	Equal to one if there a whistle-blowing mechanism allowing individuals to raise a concern about wrongdoing occurring in the pharmaceutical sector of your country.
5.01.01	Equal to one if there are legal provisions establishing the powers and responsibilities of the Medicines Regulatory Authority (MRA).
5.01.02	Equal to one if there is a MRA.
5.01.04.02	Equal to one if the MRA is a semi autonomous agency.
5.01.10	Equal to one if an assessment of the medicines regulatory system has been conducted during the five years before the survey.
5.01.11	Equal to one if the MRA gets funds from regular budget of the government.
5.01.15	Equal to one if the MRA uses a computerized information management system to store and retrieve information on registration, inspections, etc.
5.02.15S	Equal to one if legal provisions require declaration of potential conflict of interests for the experts involved in the assessment and decision-making for registration.
5.05.03	Equal to one if Good Manufacturing Practices (GMP) requirements are published by the government.
5.05.07	Equal to one if National Good Distribution Practice requirements are published by the government
5.05.11	Equal to one if National Good Pharmacy Practice Guidelines are published by the government

Access to quality medicines can only be ensured by the presence of a strong regulatory system. Control over the processes and people involved in the production, distribution, and sales of medicines requires a legal framework for licensing, monitoring quality, and pharmacovigilance. Every modern regulatory system requires a legislative framework to permit the implementation and enforcement of policies as well as ensure that relevant actors meet predetermined requirements of quality before participating in the market. Without licensing requirements the quality of medicines cannot be ensured; although licensing does not provide proof of quality, it is an indispensable element. Ongoing monitoring and quality control of all segments of the private market also plays an important role in safeguarding the quality of medicines. Not only must quality control check that products contain the right ingredients in the right amounts, it must also verify that products are transported and stored correctly, an especially important aspect of quality control in hot and humid climates. Pharmacovigilance ensures the safety of drugs consumed within the market. Given that adverse drug reactions (ADRs) are common and can often, directly or indirectly, cause death, every country should have a system in place to monitor medications for potential side-effects. The detection, assessment, understanding, and prevention of ADRs play a critical role in protecting public health.

To assess the degree to which each country regulates the private market for medicines the index of *Monitoring the Private Market* focuses on the three dimensions discussed above to construct a single indicator. Each dimension incorporates at least eight indicators. Table 2 details each of the indicators used in each of the dimensions.

**Table 2:** Composition of *Monitoring the Private Market*

Legal Framework	
5.02.08	Equal to one if medicines registration always includes the INN (International Nonproprietary Names).
5.02.12S	Equal to one if legal provisions require publication of a Summary of Product Characteristics (SPCs) of the medicines registered.
5.05.01	Equal to one if legal provisions exist requiring manufacturers to be licensed
5.05.02	Equal to one if legal provisions exist requiring both domestic and international manufacturers to comply with Good manufacturing Practices (GMP)
5.05.05	Equal to one if legal provisions exist requiring wholesalers and distributors to be licensed
5.05.06	Equal to one if legal provisions exist requiring wholesalers and distributors to comply with Good Distributing Practices
5.05.08	Equal to one if legal provisions exist requiring pharmacists to be registered
5.05.09	Equal to one if legal provisions exists requiring private pharmacies to be licensed.
5.07.01	Equal to one if legal provisions exist to control the promotion and/or advertising of prescription medicines
Inspection of Supply Chain and Distribution^a^	
5.03.05.01	Equal to one if local manufactures are inspected for GMP compliance.
5.03.05.02	Equal to one if private wholesalers are inspected.
5.03.05.03	Equal to one if retail distributors are inspected-
5.03.05.05	Equal to one if pharmacies and dispensing points of health facilities are inspected.
Inspections	Equal to one if manufactures, retail distributors, and pharmacies and dispensing points of health facilities are inspected at least every three years. Or
	Equal to one if either manufactures, retail distributors, or pharmacies and dispensing points of health facilities are inspected at least every year.
5.04.02	Equal to one if legal provisions exist allowing the sampling of imported products for testing
5.04.03	Equal to one if legal provisions exist requiring importation of medicines through authorized ports of entry
5.04.04	Equal to one if legal provisions exist allowing inspection of imported pharmaceutical products at the authorized ports of entry.
5.05.04	Equal to one if legal provisions exist requiring importers to be licensed
Pharmacovigilance	
5.10.01	Equal to one if there are legal provision that provides for pharmacovigilance activities as part of the MRA mandate.
5.10.02	Equal to one if legal provisions exist requiring the Marketing Authorization holder to continuously monitor the safety of their products and report to the MRA
5.10.03	Equal to one if legal provisions about monitoring Adverse Drug Reactions (ADR) exist in your country
5.10.22S	Equal to one if there training courses in pharmacovigilance.
5.10.05	Equal to one if an official standardized form for reporting ADRs is used.
5.10.06	Equal to one if a national ADR database exists.
5.10.10	Equal to one if there a national ADR or pharmacovigilance advisory committee able to provide technical assistance on causality assessment, risk assessment, risk management, case investigation and, where necessary, crisis management including crisis communication.
5.10.16S	Equal to one if ADR database is computerized.

^a^Several continuous variables (e.g. How many samples were taken in the last two years) were discarded due to what seemed invalid values

Especially in poorer countries citizens’ access to medicines depends on the public health system. For this reason it is critical to assess the degree and standards of quality control with the public health system. Following the recommendations of the WHO, the licensing of pharmacists, public pharmacies, and dispensing points is an important part of regulation [37]. Public pharmacies should also be routinely inspected and sampling of medicines should take place. The quality of medicines distributed by the public sector depends directly on how government agents procure them and how they are stored. Testing public products prior to acceptance and prequalifying suppliers can play a critical role in ensuring the quality of medicines by reducing corruption within the public health system. A competitive and transparent procurement process can also help safeguard the quality of medicines. Table 3 details the indicators used to develop the index *Public Quality Control*.

**Table 3:** Composition of *Public Quality Control*

5.03.05.04	Equal to one if public pharmacies and stores are inspected.
5.03.05.05	Equal to one if pharmacies and dispensing points of health facilities are inspected.
5.05.08	Equal to one if legal provisions exist requiring pharmacists to be registered.
5.05.10	Equal to one if legal provision exist requiring public pharmacies to be licensed.
5.06.04.01	Equal to one if quality monitoring in the public sector exists (routine sampling in pharmacy stores and health facilities).
5.06.04.06	Equal to one if there is testing of public program products prior to acceptance and/or distribution.
7.01.03	Equal to one if public sector requests for tender documents are publicly available.
7.01.04	Equal to one if public sector tender awards are publicly available.
7.01.05	Equal to one if there is a system to prequalify suppliers.
7.01.07S	Equal to one if there is a written public sector procurement policy.
7.01.10S	Equal to one if a process exists to ensure the quality of products procured.
7.01.11S	Equal to one if a list of samples tested during the procurement process and results of quality testing are available.
7.01.12.01S	Equal to one if tenders are national competitive.
7.01.12.02S	Equal to one if tenders are international competitive tenders.
7.02.03	Equal to one if there are national guidelines on Good Distribution Practices (GDP)
7.10.10.01S	Equal to one if the quality assurance process includes prequalification of products and suppliers.
7.10.10.02S	Equal to one if explicit criteria and procedures exist for prequalification of suppliers
7.10.10.03S	Equal to one if a list of prequalified suppliers and products is publicly available.

With our indices of *State Regulatory Infrastructure*, of *Monitoring the Private Market* as well as of *Public Quality Control* can broadly review coverage of the key dimensions of drug regulation identified by the WHO, allowing us to explore different aspects of the policies that governments have in place. The aim is to identify variation across small and developing countries by constructing indices that measure the underlying quality and character of the laws, policies, and practices in each country based on their responses to the WHO *Pharmaceutical Situation Report*. To do this we draw on item response theory (IRT) to reduce multiple observed indicators into latent variables that represent the three discrete aspects of regulation understudy. As argued above, the items selected from the survey represent what are believed to be key and often neccasry apsects of an effective regulatory system.

One of the problems with trying to measure regulatory quality is that no direct means of measurement exists. Much like the underlying quality of a doctor or the intelligence of a student, regulatory quality can only be measured indirectly through various indicators. As indicators of pharmaceutical regulatory quality, we have chosen the presence or absence of specific regulatory agencies, laws, policies, or practices identified as important by the WHO. IRT allows us to measure an underlying unobservable trait (the quality of the regulatory system) by estimating the relationship between the unobservable trait (latent variable) of interest and the probabilities of specific responses to specific items. To construct our indices, we use IRT to estimate the relationship between regulatory quality and responses to the survey questions listed in Tables 1, 2 and 3. In its simplest form, by using IRT we posit that the probability of a given country with a given regulatory quality responding positively to a given survey item is conditioned by the regulatory quality of the country and by the specific properties of the regulatory system that the item represents. By measuring the observed covariance among responses of numerous countries to items with different characteristics, ITR can discriminate between countries and estimate the underlying regulatory quality of the country. This allows IRT to discriminate between countries in the same way that a teacher uses different types of questions and questions of different difficulties to evaluate students. After all, having legal provision for the promotion of medicines is not the same as the publishing a summary of product characteristics, the former tells us much more about the underlying quality of pharmaceutical regulation. Nor is having a semi-autonomous Medicines Regulatory Authority (MRA) the same as having a MRA with guaranteed funding, in developing countries guaranteed funding is much more difficult to achieve.

Using IRT has two important benefits. First, IRT allows the data to provide the weights assigned to each item within the indices (how much each item discriminates between countries). This frees us from arbitrarily assigning weights to each question within the survey when constructing the indices. Moreover, since there is no clear theoretical or normative theory from which we can derive weights, doing so, or weighing each question equally could significantly bias the resulting indices and the ranks of the countries within it. Second, the methodology used provides greater information about the uncertainty of the final index value for each country. Rather than providing a simple point estimate, as most indices do, we acknowledge the uncertainty in our model and provide this information so that others can take it into account in their own work.

This paper uses Bayesian sampling methods to estimate and item response model for each of the proposed indices. In order to map each country’s observed responses onto the latent variable, we assume that **X** denotes a N*J matrix of observed responses to the J survey questions by N countries. We further assume that the elements of **X** are derived from a N*J matrix X* latent variables and a collection of cut points that distinguish between responses. Setting j = 1,…,J index of observed response variables and i = 1,…,N index of observations, the association between the observed values **X** and the latent values X* is modeled using a factor analysis model:

$$ {X}_i^{*}=\Lambda {\phi}_i+{e}_i\ i=1,\dots .,n $$

where Λ is a J × K matrix of discrimination scores (factor loadings), *ϕ*_*i*_is a K vector of factor scores unique to each observation i, and e_i_ represents a J vector of disturbances (see Quinn 2004 for further explanation).^7^

Using Bayesian sampling methods, we estimate an item response model for each of the three dimensions of pharmaceutical regulation identified above:

1. *State Regulatory Infrastructure*
2. *Public Quality Control*.
3. *Monitoring the Private Market* with:
  - at least regular inspections
  - at least annual inspections

## Results

The item response model estimates the underlying quality of the regulatory structure of each country (our indices: *ϕ*), for each of the three dimensions, as well as the discrimination and difficulty scores of each survey item used to construct the index. The discrimination scores measures the degree to which “better” regulatory structures have a positive score on the item. For example, whether or not a government publishes its good manufacturing practices (GMP) requirements has a particularly high discrimination score, while whether or not the MRA is semi-autonomous agency has a relative low discrimination score. This suggests that countries with high levels of *State Regulatory Infrastructure* are much more likely to publish their GMP requirements, but they are not necessarily more likely to have a semi-autonomous MRA. This does not mean that a semi-autonomous MTA does not produce better regulatory results. Rather, it suggests that both good and bad regulatory systems can have semi-autonomous MRAs. As such, the presence of a semi-autonomous MRA does not provide much information about the quality of the regulatory system.

The difficulty score measures the difficulty of each item for all of the responding countries. The difficultly score takes on both positive and negative values and suggests the likelihood that most countries response positively on the item. While we might assume that having a lot of difficult items in our index would better discriminate between countries, there is no direct relationship between difficulty scores and the level of discrimination. Often very “easy” attributes can be highly discriminatory; for example, surgical hand scrubbing is a very easy act and, at the same time, clearly discriminates between good and bad surgeons. Although the difficulty level does not have a direct association with the quality of regulation, it is important that any index constructed from numerous elements should have a range of item difficulties (Baker 2001). This allows all countries within the sample to have in place at least some of the regulatory “goals” and, therefore, allows the establishments of a meaningful lower value of the index. Less difficult items allow us to discriminate between mediocre and poor quality regulatory systems.

As can be seen in Table 4, the difficulty estimates for the fourteen items that make-up the *State Regulatory Infrastructure* index range from −0.84 to 1.38.^8^ The range of difficultly indicates that the index is composed of easy and difficult items. Although the difficulty of an item and its discriminatory value are not directly related, it is interesting to note that two of the items that were most likely to be answered positively (low difficulty scores): whether the government published National Good Pharmacy Practice Guidelines and National Good Distribution Practice Guidelines, also have high discrimination scores. In a sense, we can think of this as surgical hand scrubbing, it is an easy, but necessary step for a good regulatory system.

**Table 4:** Posterior density of the measure of *Regulatory Infrastructure and Good Practices*

	Item discrimination	Item difficulty
GMP requirements are published by the government (5.05.03)	1.89	0.08
	(0.72)	(0.30)
MRA uses a computerized information management system (5.01.15)	1.67	0.61
	(0.60)	(0.30)
Government publishes National Good Pharmacy Practice Guidelines (5.05.11)	1.50	−0.84
	(0.57)	(0.31)
Government publishes National Good Distribution Practice Guidelines (5.05.07)	1.23	−0.76
	(0.46)	(0.27)
An MRA exists (5.01.02)	1.20	1.34
	(0.42)	(0.32)
Legal provisions establish the power and responsibilities of the MRA (5.01.01)	1.14	1.38
	(0.39)	(0.31)
Whistle-blowing mechanism for the pharmaceutical sector (3.01.16)	0.95	0.04
	(0.32)	(0.20)
A national medicines policy official document exists (3.01.04)	0.77	0.78
	(0.28)	(0.20)
Declaration of potential conflict of interests of experts (5.02.15S)	0.75	−0.25
	(0.27)	(0.18)
MRA gets funds from regular government budget (5.01.11)	0.66	0.77
	(0.26)	(0.20)
Policy exists to manage and sanction conflict of interest (3.01.14)	0.56	−0.45
	(0.23)	(0.17)
An assessment of the regulatory system was conducted within last five years (5.01.10)	0.51	0.56
	(0.23)	(0.17)
Pharmaceutical policy implementation is being regularly monitored (3.01.12)	0.45	−0.06
	(0.21)	(0.16)
*MRA is a semi autonomous agency (5.01.04.02)*	*0.36*	−0.71
	*(0.23)*	(0.17)

Notes: Entries without parentheses are posterior means and entries with parentheses are posterior standard deviations. The parameters were estimated using the MCMCmixfactanal in the MCMCpack in R3.1.2. Items are ordered by the degree of discrimination. Estimations with a significant portion of their posterior mass to the left of zero are in italic and placed last

High discrimination scores are directly associated with better regulatory characteristics. Three of the variables with the highest discrimination scores have a direct relationship with the publication by the government of good practice guidelines (5.05.03, 5.05.07, and 5.05.11). The variable with the second highest discrimination scores takes into account whether the MRA uses a computerized information management system. Although the publication of guidelines or having a computerized information management system may seem less important than having a national medicines policy or a medicines regulatory authority, the higher discrimination scores of this variable should not be taken to mean that these actions (variables) are more important than other actions. The publication of these guidelines usually emerges from the implementation of a national medicines policy and a causal relationship would seem to exist between the two. However, not all countries with a national medicines policy have taken the time or have the ability to develop good practice guidelines. The discrimination scores on the variables associated with the publication of good practice guidelines can be interpreted as the benefit accrued by developing and publishing core criteria for the pharmaceutical market, after establishing a national medicines policy. At the same time, it is little use to have good policies on the books if regulators cannot easily track licenses, registration, medicines, etc. through a modern computerized information management system.

Whether the government published GMP requirements and whether the MRA uses a computerized information management system have the two highest discrimination scores. These high scores indicate that the governments with the best underlying regulatory infrastructures have these elements. On the other hand, whether the MRA is a semi-autonomous agency has a very low discriminatory value. The high standard deviation of this item indicates that it may have an “insignificant” relationship with the degree of regulatory infrastructure. In some ways this makes sense, although a semiautonomous agency may be better able to resist political pressure, it is not a guarantee that the agency and its regulators will have the legal provisions or resources necessary to regulate the highly complex pharmaceutical market.

**Fig. 1:**
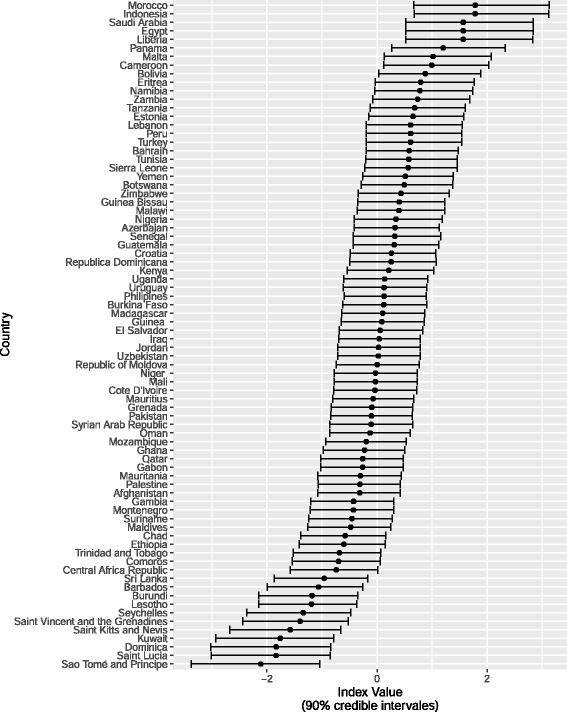
Quality of regulatory infrastructure (estimated *ϕ*)

While the discrimination and difficultly scores of the items used to construct the index provide information on the overall patterns of association among the responses within the index, they do not provide clear information about which countries have better underlying regulatory structures. One of the advantages of estimating a Bayesian model is that it provides us with point estimates (*ϕ*) for regulatory quality (the latent factor) for each country, which allows comparison across countries and groups of countries. Figure 1 presents the estimated *ϕ*s for the *State Regulatory Infrastructure* index. Interpreting the *ϕ*s as a measure of the regulatory infrastructure in the pharmaceutical sector we see that Morocco, Indonesia, Saudi Arabia and Egypt ranked highest among the countries within the survey (see Fig. 1). Among the countries with the lowest score are the Central African Republic, Lesotho, and numerous small island nations.

Estimating a Bayesian model also has the advantage of providing uncertainty estimates for our estimated *ϕ*s. Quinn (2004) points out that the marginal 90 % credibility intervals for the estimated scores can be misleading due to the positive correlation between the estimates *ϕ*s of countries [38]. To estimate the the differences that exist between the *ϕ*s of different countries, even though their credibility interval overlap, we can evaluate the probability that country *a* has a higher score than country (*ϕ*_*a*_ > *ϕ*_*b*_). For instance, in Fig. 1, the posterior probability that Morocco has a higher latent factor score (*ϕ*) than Saudi Arabia (*ϕ*_*Morocco*_ > *ϕ*_*Saudi Arabia*_) is 50 % and the probability that Morocco has a higher score than Panama is 77 %. Similarly the probability that *ϕ*_*Morocco*_ > *ϕ*_*Turkey*_=95 %, the probability that *ϕ*_*Saudia Arabia*_ > *ϕ*_*Turkey*_= 94 %, and the probability that *ϕ*_*Panama*_ > *ϕ*_*Turkey*_= 82 %.

We can also calculate the probability that a country of a group of countries have the highest (lowest) scores. In the case of Morocco, there is a 24 % probability that it has the highest score among all the countries and there is a 49 % probability that either Morocco or Indonesia have the highest score among all of the countries. Comparing Morocco and Indonesia against Saudi Arabia, Egypt, and Liberia we find a 59 % probability that either Morocco or Indonesia have a higher score than any of the three former countries. In the case of São Tomé and Principe there is a 35 % probability that the country has the lowest score of all the countries. However, when we compare São Tomé to only Dominica and Saint Lucia there is a 48 % that its score is lower than those of the other two countries.

These results may seem contrary to accepted perceptions of how well these countries regulate their pharmaceutical markets in comparison to the other countries within the survey. For example, neither Liberia nor Namibia are considered to regulate their pharmaceutical markets well [39, 40]. However, both countries have taken great strides to establish a strong regulatory infrastructure and legal framework, which would give them a high *de jure* rating. Both countries lack the resources and capacity to provide the regulatory oversight [40, 41]. These two cases highlight the importance of differentiating between *de jure* regulatory system and the overall quality of pharmaceutical regulation. In general, however, our results match much of that we know about the regulatory quality and capacity of states. For instance, both Indonesia and Saudi Arabia are known for having a strong institutional foundation for effective regulation of medicines [41–45].^9^ Similarly, the regulatory capacity of Dominica, St Kitts and Nevis, St. Lucia, and St Vincent and the Grenadines are generally considers to be very weak [46, 47].

It is interesting to note that among the 15 countries with the lowest score for *State Regulatory Infrastructure* nine are small island nations. This may indicate that the size of the country has a direct influence on the state’s capacity to regulate its pharmaceutical market.

Several factors need to be taken into consideration when interpreting these results and comparing them with other studies or our own perceptions of the regulatory quality of a specific country. First, the latent variable under consideration is not how well countries regulate their market, but whether they have “good” policies in place. There is an important difference between having regulations and policies on the books and their successful implementation. Of course, good policies are necessary for good regulation, but they are not sufficient. Second, our current understanding of how well countries regulate their markets are based on either single case studies or the comparison of a few countries, which, although they provide a rich understanding of the specific countries understudy (e.g. Ashigbie, 2010: Ratanawijitrasin and Wondemagegnehu 2002;), do not provide any means of systematically comparing regulatory structures across countries [48, 49]. The cross national study of pharmacovigilance by Olsson et al. (2010) is a good example of the lack of matrix for intra and cross country comparison [19]. Although the authors have data on pharmacovigilance for 55 developing countries no attempt is made to clearly distinguish between and evaluate the regulations and practices of countries. The text only describes the diversity of pharmacovigilance policy across countries. As such, although we may feel that we can rank countries based on case studies and small-n comparative studies, such rankings are unsystematic, may value different aspects of pharmaceutical regulation differently in different countries, and may be influenced by the quality of other aspect of the government not directly related to pharmaceutical regulation.

What is the significance of this variation for public health infrastructure? The first contribution of these indices is to show that across a group of least developed countries there are important differences in which elements of WHO pharmaceutical standards they seem to be adopting. Moreover, across a set of key policies in the *Regulatory Infrastructure and Good Practice* systems in developing countries, some policies have a much higher level of *discrimination* or *difficulty*, as illustrated in Table 4. The second contribution is to identify the specific practices associated with high regulatory standards. For example, having published Good Manufacturing Practices is highly discriminatory. The adoption and publication of Good Manufacturing Practices, in and of itself, does not guarantee any level of pharmacological adherence. What our results show is that the countries that make this step have a proclivity toward a better regulatory system in general and system harmonization. By contrast, public quality procurement systems seem to be stronger and more harmonized when they adopt prequalification systems, as illustrated in the results in Table 5.

**Table 5:** Posterior density of the measure of *Public Quality Control* (all countries)

	Item discrimination	Item difficulty
Prequalification of products and suppliers (7.10.10.01S)	3.69	1.35
	(1.05)	(0.59)
Explicit criteria and procedures exist for prequalification of suppliers (7.10.10.02S)	3.63	0.49
	(1.05)	(0.51)
Process exists to ensure the quality of products procured (7.01.10S)	2.56	2.87
	(0.83)	(0.79)
System to prequalify suppliers (7.01.05)	1.42	0.50
	(0.41)	(0.25)
List of prequalified suppliers and products is publicly available (7.01.10.03S)	1.41	−0.49
	(0.46)	(0.26)
Results of quality testing are available (7.01.11S)	1.22	−0.14
	(0.36)	(0.22)
Written public sector procurement policy (7.01.07S)	0.80	0.66
	(0.26)	(0.19)
Public sector requests for tender are publicly available (7.01.03)	0.70	1.00
	(0.26)	(0.21)
Testing of public program products prior to distribution (5.06.04.06)	0.43	0.44
	(0.20)	(0.16)
*Pharmacists required to be registered (5.05.08)*	*0.40*	1.53
	*(0.27)*	(0.24)
*Quality monitoring in the public sector exists (5.06.04.01)*	*0.31*	0.51
	*(0.17)*	(0.16)
*Pharmacies are inspected (5.03.05.05)*	*0.29*	0.75
	*(0.20)*	(0.17)
*Tenders are nationally competitive (7.01.12.01S)*	*0.28*	0.32
	*(0.19)*	(0.15)
*International competitive tenders (7.01.12.02S)*	*0.25*	0.58
	*(0.19)*	(0.16)
*Public sector tender awards are publicly available (7.01.04)*	*0.24*	0.50
	*(0.19)*	(0.15)
*Public pharmacies and stores are inspected (5.03.05.04)*	*0.19*	0.62
	*(0.19)*	(0.16)
*National guidelines on GDP exist (7.02.03)*	*0.14*	−0.27
	*(0.18)*	(0.15)
*Public pharmacies must be licensed (5.05.10)*	*−0.04*	0.26
	*(0.18)*	(0.15)

Notes: Entries without parentheses are posterior means and entries with parentheses are posterior standard deviations. The parameters were estimated using the MCMCmixfactanal in the MCMCpack in R3.1.2. Items are ordered by the degree of discrimination. Estimations with a significant portion of their posterior mass to the left of zero are in italic and placed last

Similar to the other indices, the items in the *Public Quality Control* index have a wide range of difficulty scores (between −0.49 and 2.87). Yet, the results of this index are particularly weighted toward one aspect of public regulation. Four of the items with the greatest discrimination scores are associated with the prequalification of products and suppliers. Countries that adopt these norms have better regulatory systems and are more adherent to the norms set by the WHO. This is a very interesting result, because it confirms that the past decade of work with WHO as an interlocutor for developing countries in the purchase and distribution of medicines (e.g. through the Global Fund to Fight AIDS, Tuberculosis and Malaria) has created an incentive for countries to adhere to this norm.

Does the implementation of a prequalification program result in countries advancing other aspects of public pharmacological regulation? Or is it that by implementing other measures prequalification is easier to adopt? A clue may be found in the fact that *Public sector requests of tender are publically available* is the only aspect of the public tender process that discriminates between countries. It may seem strange that whether tenders are competitive is non-discriminatory, but whether tender awards are publicly available does discriminate between countries. However, public requests for tender is a necessary condition for any truly competitive tender process. The lower difficulty scores for *Tenders are nationally competitive* and *International competitive tenders* indicates that many countries respond positively to these questions even though they do not make requests for tender publically available, which would significantly limit the degree of competitiveness of any tender request. Announcing tender requests publically limits the ability of government offices to target specific companies for tender either in an attempt to promote national companies or in return for kick-backs. The implementation of a strict prequalification program, in a sense, duplicates part of the competitive tender process, but ensuring and setting transparent products standards. A property run prequalification program many not guarantee the best prices, but it should ensure that quality medicines reach the public.^10^

The results of our third index, *Regulation and Monitoring of the Private Market* are illustrated in Table 6. The items in this index have a broad range of difficulty scores (between −0.60 and 2.29) giving us confidence that the index has meaningful upper and lower values. Within these results, legal provisions (LP) play a key role in discriminating between countries. The four items with the highest discrimination scores directly measure the presence of legal provision for the promotion and manufacture of medicines as well as for pharmacovigiliance activities by the MRA. Legal provisions are often an under-recognized aspect of effective pharmaceutical regulation. Although regulations provide policymakers with more flexibility, legislation established the foundation and framework for good regulatory measures. As suggested by the results in Table 6, inspecting manufactures has little benefit if regulators do not have the legal authority to conduct inspections or sanction infractions.

**Table 6:** Posterior density of the measure of the regulation and monitoring of the private market model

	Aspect	Annual inspections		Regular inspections	
		Item discrimination	Item difficulty	Item discrimination	Item difficulty
Legal provisions for the promotion of medicines (5.07.01)	LF	2.21 (0.70)	2.02 (0.55)	2.15 (0.68)	1.98 (0.54)
Manufactures required to be licensed (5.05.01)	LF	2.06 (0.68)	2.29 (0.59)	2.05 (0.67)	2.28 (0.58)
Manufactures must comply with GMP (X5.05.02)	LF	1.99 (0.62)	1.54 (0.43)	1.97 (0.61)	1.53 (0.43)
Pharmacovigilance part of MRA mandate (5.10.01)	PV	1.75 (0.48)	0.32 (0.28)	1.75 (0.47)	0.31 (0.28)
Private wholesalers inspected (5.03.05.02)	SC	1.74 (0.56)	1.36 (0.39)	1.81 (0.58)	1.40 (0.41)
Local manufactures inspected for GMP compliance (5.03.05.01)	SC	1.72 (0.51)	0.72 (0.31)	1.77 (0.53)	0.73 (0.31)
Retail distributors inspected (5.03.05.03)	SC	1.69 (0.54)	1.36 (0.38)	1.75 (0.56)	1.37 (0.40)
Legal provisions for ADR (5.10.03)	PV	1.62 (0.46)	0.05 (0.26)	1.58 (0.45)	0.05 (0.25)
Wholesalers and distributors must be licensed (5.05.05)	LF	1.52 (0.54)	2.15 (0.50)	1.50 (0.53)	2.13 (0.49)
Medicines registration always includes the INN (5.02.08)	LF	1.25 (0.39)	1.19 (0.29)	1.20 (0.38)	1.16 (0.28)
Official standardized form for reporting ADRs is used (5.10.05)	PV	1.17 (0.37)	1.26 (0.30)	1.16 (0.36)	1.26 (0.29)
National ADR database exists (5.10.06)	PV	1.13 (0.31)	0.07 (0.21)	1.10 (0.31	0.07 (0.21)
Marketer monitor safety and report to the MRA (5.10.02)	PV	1.11 (0.33)	0.05 (0.21)	1.09 (0.32)	0.05 (0.21)
National ADR or pharmacovigilance advisory committee (5.10.10)	PV	1.09 (0.33)	−0.60 (0.22)	1.10 (0.33)	−0.60 (0.23)
Importers required to be licensed (5.05.04)	SC	1.02 (0.43)	1.92 (0.39)	0.98 (0.42)	1.89 (0.38)
Points of dissemination inspected (5.03.05.05)	SC	0.94 (0.31)	0.90 (0.23)	0.97 (0.31)	0.91 (0.23)
Pharmacies are required to be registered (5.05.09)	LF	0.89 (0.30)	0.87 (0.23)	0.88 (0.29)	0.86 (0.22)
ADR database is computerized (5.10.16S)	PV	0.87 (0.26)	−0.09 (0.19)	0.87 (0.26)	−0.10 (0.19)
Training courses in pharmacovigilance exist (5.10.22S)	PV	0.85 (0.27)	0.41 (0.19)	0.86 (0.27)	0.41 (0.19)
Distributors must comply with GDP (5.05.06)	LF	0.82 (0.26)	−0.02 (0.18)	0.80 (0.25)	−0.02 (0.18)
Sampling of imported products for testing (5.04.02)	SC	0.55 (0.23)	0.64 (0.18)	0.53 (0.23)	0.63 (0.18)
Publication of SPCs of registered medicines (5.02.12S)	LF	0.46 (0.21)	−0.07 (0.16)	0.47 (0.21)	−0.07 (0.16)
Regularity of inspections	SC	0.33 (0.11)	−0.38 (0.16)	0.63 (0.25)	−0.79 (0.19)
*Importation only through authorized ports (5.04.03)*	SC	*0.42 (0.21)*	0.54 (0.17)	0.41 (0.21)	0.54 (0.17)
*Pharmacists required to be registered (5.05.08)*	LF	*0.17 (0.27)*	1.43 (0.22)	0.14 (0.26)	1.42 (0.22)
*Inspection of imported pharmaceutical products at ports of entry (5.04.04)*	SC	*0.14 (0.20)*	0.35 (0.15)	0.12 (0.19)	0.35 (0.15)

Notes: Entries without parentheses are posterior means and entries with parentheses are posterior standard deviations. The parameters were estimated using the MCMCmixfactanal in the MCMCpack in R3.1.2. Items are ordered by the degree of discrimination. Estimations with a significant portion of their posterior mass to the left of zero are in italic and placed last. Aspects of monitoring the private market are: Legal Framework (LF), Control and Inspection of the Supply Chain (SC), and Pharmacovigilance (PV)

The results shown in Table 6 indicate that regulatory systems among developing countries appear to be surging toward harmonization with international standards through regulation of companies as they register and introduce their products in national markets. Better regulatory systems are not those with consumer-level regulation (through pharmacists or pharmacies) nor international trade-level regulation (through ports). Better regulation is indicated by licensing permission to companies, compliance with GMP, and pharmacovigilance, with scant emphasis on how products ate dosseminated. For example, we find that whether inspections are conducted on a regular basis (*Regularity of Inspections*) as well as *Importation only through authorized ports* and *Inspection of imported pharmaceutical products a port of entry* have little discriminatory value. Interestingly, restricting ports of entry and conducting inspections at the port are an important part of the regulatory arsenal in industrialized countries, indicating that developing countries may be developing their own regulatory norms.^11^

**Fig. 2:**
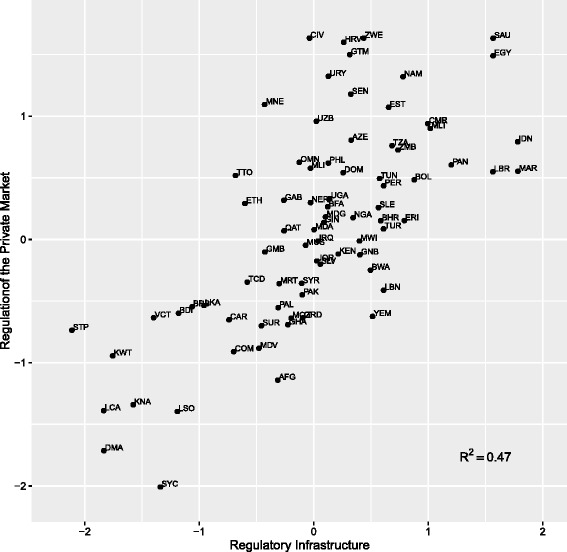
Relationship between regulatory infrastructure and regulation of the private market

When we examine how these variables play out across our set of countries, the results may seem counter-intuitive. Figure 2 presents the estimated scores for each country for the *Regulation and Monitoring of the Private Market* contrasted with *State Regulatory Infrastructure*. Saudi Arabia (SAU), Zimbabwe (ZWE), and Cote d’Ivoire (CIV) share the highest scores for the regulation of the private market.^12^ At the same time, these three countries have very different values for *State Regulatory Infrastructure*. While this indicates that there is not a direct relationship between *State Regulatory Infrastructure* and the *Regulation and Monitoring of the Private Market*, the empty triangle in the upper left-hand corner of the figure suggest that high quality regulatory infrastructure is a necessary condition for the regulation of the private market. As such, among countries with the lowest scores for private market regulation, we find many of the same small island countries that had very low values for *Regulatory Infrastructure*. These include, but are not limited to Saint Lucia, Seychelles, and Dominica. Among the scoring countries, we also find the Central African Republic and Lesotho.

Given the importance of a good legal framework and institutional mandates for the regulation of the pharmaceutical market, it is not surprising to see a positive relationship between *Regulatory Infrastructure* and the *Regulation of the Private Market*. The coefficient of determination (R^2^ = 0.48) between the two indices indicates a direct relationship between regulatory infrastructure and the regulation of the private market. Almost half of the observed variation in the *Regulation of the Private Market* can be explained by the quality of the regulatory infra-structure of the country.

**Fig. 3:**
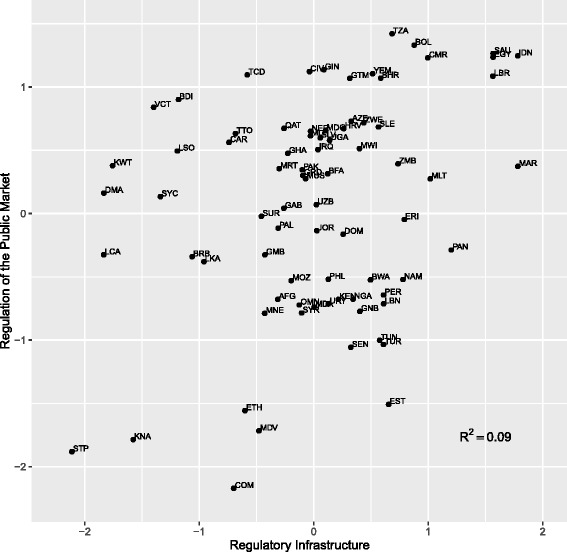
Relationship between regulatory infrastructure and regulation of the public market

Unlike the *Regulation of the Private Market*, there seems to be now relationship between rating for the *Public Quality Control* and the quality of a country’s regulatory infrastructure. Numerous countries that rated very poorly on the regulation of the private market (e.g. Dominica, Lesotho, Saint Lucia) have fairly high rating for the *Public Quality Control*. The disjuncture between *Public Quality Control* and *Regulatory Infrastructure* can be clearly seen in Fig. 3. The low coefficient of determination (R^2^ = 0.09) between the two indices, does not mean that no relationship exists between them. If we ignore Estonia, it would seem that *Regulatory Infrastructure* places a lower limit on public quality control (e.g. a sufficient condition). This is most likely because countries that have taken the time to develop the legal and institutional underpinning for pharmaceutical regulation would not find it difficult to develop and implement policies to guarantee the quality of publically procured medicines. Especially for countries that lack a domestic pharmaceutical industry, the quality of medicines can be significantly enhanced with relatively little effort by simply requiring that all medicines and suppliers have been certified by the WHO Prequalification Program.

**Fig. 4:**
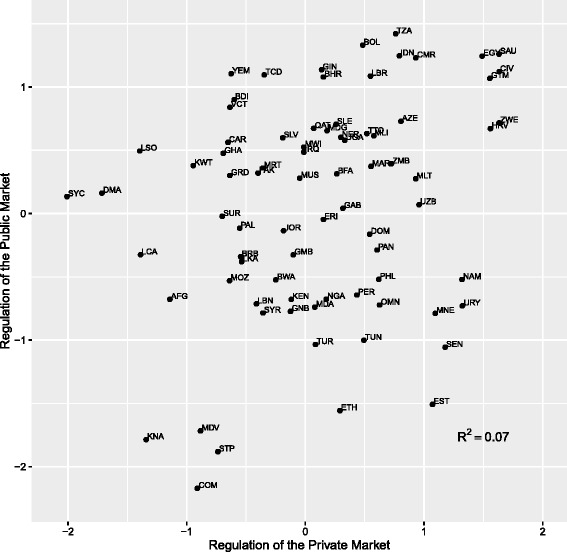
Relationship between regulation of the public and private markets

As can be seen in Fig. 4, *Public Quality Control* and *Regulation of the Private Market* have no clear association. The disjuncture between these two indices may stem from various factors. First, countries with large private markets for medicines, especially, essential medicines, may find a need to carefully regulate private distributors and retailers, while countries that provide most of their citizen’s needs through public sources may feel little need to closely regulate the private market. This would generate a negative relationship between the indicators in some countries. However, many countries have robust public and private markets for medicines (e.g. Bolivia, Dominican Republic, and Guatemala), which would require regulators to play attention to both markets. This would generate to clear relationship between the two indices. Second, the skills and regulation need to regulate one market are not the same for the other and it is unclear that significant spillover effects would occur. Moreover, as mentioned before, high levels of quality control within the public sector can be obtained through the application of prequalification programs. However, guaranteeing the provision of high quality medicines in the private market is much more difficult. Regulating the private market requires the ability and legal infrastructure necessary to inspect and sanction producers, distributors, and retailors as well as monitor a wide variety of medicines from multiple sources for adverse effects.

## Discussion

Nearly two decades on from the harmonization of global intellectual property standards, how similar are drug regulation systems globally? What policies and procedures signal higher levels of regulatory quality? This paper has set out to understand and examine variation in pharmaceutical regulation within and across small and developing countries through the construction of three indices drawing on World Health Organization surveys of pharmaceutical oversight. We use this data from 78 countries to estimate and analyze the regulatory quality of countries through item response theory. This allows us to uncover patterns within and across countries to explore which have moved toward one standard for market oversight. The purpose of these indices is not to evaluate what causes specific regulatory regimes to emerge or what causes variation within and between states, but to demonstrate and explore patterns in the variation of regulatory practices. Three indices: *State Regulatory Infrastructure*, *Monitoring the Private Market* and *Public Quality Control*, provide a means of understanding the differences in the infrastructure necessary for pharmaceutical regulation as well as how well governments regulate their public and private pharmaceutical markets.

Our paper finds extraordinary depth and variance across global pharmaceutical systems. While it should be repeated that we do not track these systems overtime, by weighing the covariance of over 4000 data points, we can see that while global intellectual property rights systems lean toward one direction, regulatory systems in small and developing countries are highly differentiated. The variance in *Regulatory Infrastructure* is clearly seen in Fig. 1 and the variance in how countries regulate their public and private markets is evidenced in Fig. 3. If the harmonization of intellectual property rights were having significant spillover effects on pharmaceutical regulation, we might expect greater convergence among countries.

Yet, within the diversity of these systems, some patterns do emerge. Having established that variance is wide, the first finding is the strong correlation between regulatory infrastructure and regulation of the private market. Interestingly, our second observation is a lack of relationship between regulatory infrastructure and the public market. Finally, we find that quality regulation of the public market is highly correlated with the prequalification of suppliers.

The fact that the prequalification of suppliers and related polices discriminate among public systems is interesting. In rough terms, this result suggests that you can rank the regulation of public medicine systems by whether and how they manage the prequalification of suppliers. This does not mean that monitoring the public sector, inspecting public pharmacies, or having international tenders are not worthwhile policies. Rather, the results indicate that both good and bad systems have these policies and what marks a truly good public medicines system is obtaining quality products in the first place.

Because the WHO has had as a keystone of its drug distribution programs a prequalification system, the results provide evidence of the diffusion of global standards through norms-making institutions such as an international agency. They also suggest that encouraging and supporting countries in the adoption of prequalification programs has been beneficial to quality of public systems in small and developing countries. But if there has been headway in prequalification programs, it must be said that limits are clear. Quality regulation does not have feedback across public and private markets and sometimes is cross-matched. Our findings demonstrate no relationship between the adoption of global standards for public pharmaceutical markets and for private markets. That is to say, states that establish rules for transparency of standards in their private pharmaceutical markets are no more likely to set standards for public purchasing systems and vice versa. For example, our results suggest that compared across the cohort, Lesoto is very good at regulating the public market but not very good at regulating the private market. By contrast, Estonia is good at regulating the private market but not good at regulating the public market. This seems consistent with the narrative of medical system development in these countries. Lesoto has been shaped by exogenous interventions, its public market emerged out of co-purchases with international institutions which demanded prequalification standards. Estonia and other Eastern European states were strongly shaped by speedy privatization and private market change in the wake of the transition from communism [50]. This paper works to see the forest for the trees, but we are acutely aware that each tree has been shaped by its unique particularities of sun and soil. We must work to understand how regulatory systems in developing countries are evolving and what impact they might have on access to medicine for its citizens.

The results presented here make an important contribution to our understanding of patterns in global regulatory diffusion. Scholarship in this area has emphasized two models for norm expansion. One suggests that the key variable in the expansion of global norms is through regional mechanisms. Another is that important global players establish rules that then are, either through processes of “closeness” to global norm builders [51]. The underlying causes of “bureaucratization” or “rationalization” of the state apparatus as DiMaggio and Powell so famously described regulatory standardization in the 1980s [52] appears in the case of the globalization of pharmaceutical standards to have far less reach. Instead our findings suggest that there is little evidence of developing countries moving toward one global pharmaceutical norm. Across this sample of countries, important variations remain across all indices. This is puzzling given that global pharmaceutical norms for intellectual property standards have reshaped the rules form ownership of drugs in developing counties [53].^13^ That the countries surveyed here have harmonized their pharmaceutical sectors to a global norm for patent rights, but resisted to either instate basic standards for pharmaceutical product entry and oversight is an interesting anomaly in story of health norms globalization. These results call for more research to unravel why there is state resistance or adherence to emerging regulatory norms and what implications of significant inter and inter-country variations in the regulation of pharmaceuticals might have for global health standards, open pharmaceutical markets, and access to medicines of quality standards to citizens of the developing world?

## Conclusions

In this paper, we offer three Indices of pharmaceutical market oversight, cross-weighted to allow for the most inclusive global study (we are aware of) comparing 78 small and developing countries with regulatory data collected by the World Health Organization (WHO). To build our Indices, we draw on item response theory (IRT) to reduce multiple observed indicators into latent variables that represent the three discrete aspects of regulation. The elements included from the survey are widely held to be the best practices in an effective regulatory system. How widely held are these practices? Our metrics allow us to breakdown and compare pharmaceutical market regulation across publics and private market monitoring systems. The first index measures the existence of basic “State Regulatory Infrastructure.” The second index assesses the degree of statutory control over private transactions: “Monitoring the Private Market”. The third index, “Public Quality Control,” evaluates the existence of polices associated with high standards of control of the public pharmaceutical market. No indicator can be a complete reflection of an oversight system. Like the Ginarte and Park [54, 55] index of intellectual property, our instrument does not endeavor to illustrate how well systems are enforced, but it is a marker of how regulatory systems are moving toward one model or splintering into diverse systems.

Building on the Indices, our paper concludes with three main results: First, we show tremendous global variation. Global pharmaceutical standards are in a moment of fracture. In contrast to the nearly complete harmonization of legal systems for patent ownership at a global level, a universal norm for pharmaceutical legislation is yet to emerge. Second, when there are changes to systems, they appear to occur in starts and fits. There is no smoothness across public and private markets. Some countries have private markets at a more advanced regulatory states and others lead their market reform and oversight from their public sectors. As shown in the contrasting cases of Lesotho and Estonia, reforms of pharmaceutical markets are part of a story both of the public (and sometimes global) effort to eliminate disease. It can also be a story of liberalization and deregulation. That Eastern Europe both came out of a transition from a communist market system, as deeply affected by the financial crisis of 2008, and now living in the instability of European Union speaks to the place of medicine at a cross-roads of different regulatory systems and institutions.

Finally, our research re-enforces the need for more comprehensive and comparable inter-temporal information on the regulation of pharmaceutics. Little evidence-based research has been conducted on the variation of regulation and norms within and across countries, in part, because the data is not available. By estimating the first cross-national Indices of pharmaceutical regulation we provide scholars and policymakers the means to evaluate a key public health policy in developing countries. We believe these Indices offer promise and will be criticised, improved upon and tested. But we are also very aware that the scope of our index is limited by the lack of available data. Without better data, we cannot fully understand how regulatory norms emerge in the pharmaceutical sector, why significant variation persists within and between countries, or in what areas specific groups of countries are falling behind. This research belongs in a stream of empirical, data-driven evidence showing that setting of rules and norms is critical to protecting the interests of public health.

## Additional file

Additional file 1: Country list and additional figures. (PDF 1.04 MB)

## Abbreviations

ADR: Adverse drug reaction
EMA: European Medicine’s Agency
GMP: Good manufacturing practices
IRT: Item response theory
MRA: Medicine regulatory author
TRIPS: Trade-related aspects of intellectual property rights
WTO: World Trade Organization

## Footnotes

1 A recent exception can be found in a quasi-experimental study conducted in Sarajevo which surveyed the clinical decision to administer drugs for critically ill patients evaluating of cost and quality [56].

2 A select number of case studies offer insights into particular countries, but there are, as we know of at this time, no global or regional-level quantitative studies. For a recent study on the structures underpinning the Japanese generic market, see Jakovljevic et al. [57].

3 Important work on this can be found in a decade-old review of generic policies in Latin America by Homedes, Linares and Ugalde which finds significant variation in the mere definition of what precisely is a generic pharmaceutical product [58].

4 Country situation reports were downloaded from http://www.who.int/medicines/areas/coordination/coordination_assessment/en/index1.html. Additional file 1 has a list of countries used in this study.

5 The authors recognize that different administrative traditions may have different approaches to resolving the same problem and the indicators may miss assess certain aspects of a government’s policies. This is a problem with almost all large scale indicators.

6 The datasets supporting the conclusions of this article are available in the Harvard dataverse repository, found at https://dataverse.harvard.edu/dataset.xhtml?persistentId=doi:10.7910/DVN/KOM12J.

7 Bayesian sampling methods are implemented by a Markov Chain Monte Carlo (MCMC) model using the MCMCpack in R [38]. We constrain one item to have a positive loading. To ensure convergence and stationary of all estimated parameters, after discarding the initial 60,000 iterations as a burn-in, we run at least 15,000,000 iterations and keep every 100th scan in our posterior sample. Two dimensional latent trait models were also estimated, but they provided no additional information and are not presented here.

8 Whether the government publishes National Good Pharmacy Practices Guidelines (5.05.11) and whether legal provisions establish the powers of the MRA (5.01.01) respectively represent the lower and upper values.

9 The low *Regulatory Infrastructure* scores for Kuwait, Oman, and Qatar may be the result f the Gulf Cooperation Council deciding to institute a centralized regulatory system in 2009, which is obsessively dominated by Saudi Arabia.

10 Given that many developing countries simply accept the pre-qualification results of developed countries or those of the WHO, one could argue that having a pre-qualification program provides no evidence of having a strong national regulatory system.

11 One notable example was the controversial port seizure of generic HIV/AIDS medicine destined for Africa, withheld by the Netherlands spurring a wave of public and diplomatic outcry. The importance of ensuring access to anti-retroviral medicine has been discussed by Wang et al. [59].

12 There is a 48 % probability one of these countries has the highest latent value among all 78 countries.

13 We are keenly aware that other pressures beyond harmonization of IPR have reshaped the market for medicine in developing countries in recent years. Recent work on the Serbian market for example, has shown how macroeconomic changes have affected the supply of effective drugs only to be further riled by the global financial crisis [21, 47].

## References

[1] Sell S (1995). Intellectua1 property protection and antirust in the developing world: Crisis, coercion and choice. Int Organ.

[2] Sell S (2003). Private power, public law: the globalization of intellectual property rights.

[3] Jawara F, Kwa A (2003). Behind the scenes at the WTO.

[4] Haakonsson SJ, Richey LA (2007). TRIPs and public health: the Doha Declaration and Africa. Development Policy Review.

[5] Matthews D (2004). WTO decision on implemenationa of paragraph 6 on the DOHA declaration on the TRIPS agreement and public health. J Int Econ Law.

[6] Fink C, Reichenmiller P, Newfarmer R (2006). Tightening TRIPS: intellectual property provisions of U.S. free trade agreements. Trade, Doha, and development: a window into the issues.

[7] Roffe P. Bilateral agreements and a TRIPS-plus world: the Chile-USA Free Trade Agreement. QIAP. 2004;http://www.quno.org/sites/default/files/resources/FTAs-TRIPS-plus-English_0.pdf

[8] Shadlen K. International change and national responses: social coalitions and patent politics in Latin American in the 1990s. Prepared in preparation for the Meetings of the Latin American Political Economy Network. 2014; Santiago, Chile

[9] Shadlen K. Patent politics: the political economy of intellectual property rights in Latin America. Presented at the annual meeting of the International Studies Association. Montreal, Quebec, Canada 2004

[10] Deere C (2009). The implementation game: the TRIPS agreement and the global politics of intellectual property reform in developing countries.

[11] Abbott FM, Dukes G (2009). Global pharmaceutical policy.

[12] Beall R, Kuhn R (2012). Trends in compulsory licensing of pharmaceuticals since the Doha declaration: a database analysis. PLoS Med.

[13] Sampat B, Shadlen K. The form and effectiveness of policies to limit secondary pharmaceutical patents in Brazil and India. Annual Meeting of the American Political Science Association. 2013; August 29–September 1

[14] Correa CM (2011). Pharmaceutial innovation, incremental patenting and compulsory licensing. South Centre Research Paper.

[15] Chorev N (2015). Narrowing the gaps in global disputes: the case of counterfeits in Kenya. Stud Comp Int Dev.

[16] Gervais D (2008). The TRIPS agreement: drafting history and analysis.

[17] Correa CM (2000). Intellectual property rights, the WTO and developing countries: the TRIPS agreement and policy options.

[18] Sweet C. Comparative health systems: emerging generics regulations in Latin America. Presented at the Midwest Political Science Association, Currently under review. 2014.

[19] Olsson S, Pal SN, Stergachis A, Couper M (2010). Pharmacovigilance activities in 55 Low- and middle-income countries: a questionnaire-based analysis. Drug Saf.

[20] Ngwainmbi EK. Healthcare management strategy, communication and development challenges and solutions in developing countries. Lanham, Maryland. 2014; Lexington Books

[21] Tobar F, Drake I, Martich E (2012). Alternativas para la adopción de políticas centradas en el acceso a medicamentos. Revista Panamericana de Salud Pública.

[22] Kaplan W, Ritz LS, Vitello M, Wirtz V (2012). Policies to promote use of generic medicines in low and middle income countries: A review of published literature, 2000–2010. Health Policy..

[23] Jakovljevic M, Lazarevic M, Milovanovic O, Kanjevac T (2016). The new and old Europe: east-west split in pharmaceutical spending. Front Pharmacol.

[24] Jordana J, Levi-Faur D (2012). The institutional development of Latin American regulatory state. Handbook on the politics of regulation.

[25] Weyland K (2007). Bounded rationality and policy diffusion: social sector reform in Latin America.

[26] Shadlen K (2012). The Mexican exception: patents and innovation policy in a non-conformist and reluctant middle income country. Eur J Dev Res.

[27] Carpenter D. Reputation, Information and Confidence – The Political Economy of Pharmaceutical Regulation. Essay for Farber and O’Connell Public Choice and Public Law Volume. 2010

[28] Nunn AS, Fonseca EM, Bastos F, Gruskin S (2009). AIDS treatment in Brazil: impacts and challenges. Health Aff.

[29] Teixeira PR, Vitoria MA, Barcarolo J (2003). The Brazilian experience in providing universal access to antiretroviral therapy. Economics of AIDS and access to HIV/AIDS care in developing countries.

[30] Orsi F, Hasenclever L, Fialho B, Tigre P, Coriat B (2003). Intellectual property right, anti-AIDS policy and generic drugs: lessons from the Brazilian Public Health Program. Economics of AIDS and access to HIV/AIDS care in developing countries, issues and challenges.

[31] Cassier M, Correa M, Coriat B (2008). Scaling-up and reverse engineering: acquisition of industrial knowledge by copying drugs in Brazil. The political economy of HIV/AIDS in developing countries: TRIPS, public health systems and free access.

[32] Possas CA, Coriat B (2008). Compulsory licensing in the real world: the case of ARV drugs in Brazil. The political economy of HIV/AIDS in developing countries.

[33] Sweet C, Lofgren H, Williams O (2013). The political economy of pharmaceuticals in Brazil. Global pharmaceutical politics and production.

[34] Pal SN, Dodoo A, Mantel A, Olsson S, World Health Organization (2011). Pharmacovigilance and safety of medicines. The World medicines situation 2011.

[35] Nolan P (2001). China and the global business revolution.

[36] Grace C (2005). A briefing paper for DFID: update on china and India and access to medicines.

[37] WHO, UNAIDS, UNICEF (2009). Towards universal access: scaling up priority HIV/AIDS interventions in the health sector.

[38] Quinn KM (2004). Bayesian factor analysis for mixed ordinal and continuous responses. Polit Anal.

[39] Diack A, Seiter A, Hawkins L, Dweik IS. Assessment of governance and corruption in the pharmaceutical sector – lessons learned from low and middle income countries. Washington, DC: Health, Nutrition, and Population Family (HNP) of the World Bank; 2010

[40] Anisfeld M, An L, Mbaziira N, Kagoya H (2014). Sagwa. Strengthening the capacity of the Namibia Medicines Regulatory Council in the regulation of antiretroviral medicines and other essential pharmaceuticals in Namibia.

[41] Seiter A (2010). A practical approach to pharmaceutical policy.

[42] Al-Essa RK, Al-Rubaie M, Walker S, Salek S (2015). Pharmaceutical regulatory environment: challenges and opportunities in the Gulf region.

[43] Chee G, Borowitz M, Barraclough A (2009). Private sector health care in Indonesia.

[44] Bank W (2009). Pharmaceuticals: why reform is needed. Indonesia health sector review: policy and discussion notes.

[45] World Bank (2009). Pharmaceuticals: why reform is needed. Indonesia health sector review: policy and discussion notes.

[46] SHOPS. Strengthening health outcomes through the private sector and health systems 20/20: Dominica Health Systems and Private Sector Assessment. Health Systems 20/20 and SHOPS 2012

[47] WHO (2011). World medicines situation report 2011.

[48] Ashigbie PG. Pharmaceutical regulation: A twelve country study. Boston University, School of Health (Accessed 10 June 2016) Available from http://apps.who.int/medicinedocs/documents/s18042en/s18042en.pdf. 2010.

[49] Ratanawijitrasin S, Wondemagegnehu E (2002). Effective drug regulation. A multicountry study Geneva.

[50] Jakovljevic M, Djordjevic N, Jurisevic M, Jankovic S (2015). Evolution of the Serbian pharmaceutical market alongside socioeconomic transition. Expert Rev Pharmacoecon Outcomes Res.

[51] Carpenter D, Chattopadhyay J, Moffitt S, Nall C (2011). The complications of controlling agency time discretion: FDA review deadlines and postmarket drug safety. Am J Polit Sci.

[52] DiMaggio PJ, Powell WW (1983). The iron cage revisited: institutional isomorphism and collective rationality in organizational fields. Am Sociol Rev.

[53] Deere-Birkbeck C (2011). Global governance in the context of climate change: the challenges of increasingly complex risk parameters. Int Aff.

[54] Ginarte J, Park W (1997). Determinants of patent rights: A cross-national study. Research Policy..

[55] Park WG (2008). International patent protection: 1960–2005. Res Policy.

[56] Vukovic M, Gvozdenovic BS, Rankovic M, McCormick BP, Vukovic DD, Gvozdenovic BD (2015). Can Didactic Continuing Education Improve Clinical Decision Making and Reduce Cost of Quality? Evidence From a Case Study. Journal of Continuing Education in the Health Professions..

[57] Jakovljevic M, Nakazono S, Ogura S (2014). Contemporary generic market in Japan – key conditions to successful evolution. Expert Rev Pharmacoecon Outcomes Res.

[58] Homedes N, López Linares R, Ugalde A. Generic drug policies in Latin America. Health, Nutrition and Population (HNP), Discussion Paper, World Bank. 2005.

[59] Wang H, Wolock TM, Carter A, Nguyen G, Kyu HH, Gakidou E (2016). Estimates of global, regional, and national incidence, prevalence, and mortality of HIV, 1980–2015: the Global Burden of Disease Study 2015. The Lancet HIV.

