# Supplementary material for: Global pharmaceutical regulation: the challenge of integration for developing states
Source: Global Health. 2016 Dec 20;12:85. doi: 10.1186/s12992-016-0208-2 (PMC5175325; doi:10.1186/s12992-016-0208-2)

Countries Used in Study and Country Codes

Country	Code	Country	Code
Afghanistan	AFG	Malta	MLT
Azerbaijan	AZE	Mauritania	MRT
Bahrain	BHR	Mauritius	MUS
Barbados	BRB	Montenegro	MNE
Bolivia	BOL	Morocco	MAR
Botswana	BWA	Mozambique	MOZ
Burkina Faso	BFA	Namibia	NAM
Burundi	BDI	Niger	NER
Cameroon	CMR	Nigeria	NGA
Central Africa Republic	CAR	Oman	OMN
Chad	TCD	Pakistan	PAK
Comoros	COM	Palestine	PAL
Cote D'Ivoire	CIV	Panama	PAN
Croatia	HRV	Peru	PER
Dominica	DMA	Philippines	PHL
Egypt	EGY	Qatar	QAT
El Salvador	SLV	Republic of Moldova	MDA
Eritrea	ERI	Dominican Republic	DOM
Estonia	EST	Saint Kitts and Nevis	KNA
Ethiopia	ETH	St. Lucia	LCA
Gabon	GAB	St. Vincent &the Grenadines	VCT
Gambia	GMB	Sao Tomé and Principe	STP
Ghana	GHA	Saudi Arabia	SAU
Grenada	GRD	Senegal	SEN
Guatemala	GTM	Seychelles	SYC
Guinea	GIN	Sierra Leone	SLE
Guinea Bissau	GNB	Sri Lanka	LKA
Indonesia	IDN	Suriname	SUR
Iraq	IRQ	Syrian Arab Republic	SYR
Jordan	JOR	Tanzania	TZA
Kenya	KEN	Trinidad and Tobago	TTO
Kuwait	KWT	Tunisia	TUN
Lebanon	LBN	Turkey	TUR
Lesotho	LSO	Uganda	UGA
Liberia	LBR	Uruguay	URY
Madagascar	MDG	Uzbekistan	UZB
Malawi	MWI	Yemen	YEM
Maldives	MDV	Zambia	ZMB
Mali	MLI	Zimbabwe	ZWE

Index of Regulation of Pharmaceutical Market by Small and Developing Countries
(Average φ)

	Regulatory Infrastructure	Private Market Regulation	Public Market Regulation
Afghanistan	-1.14	-1.14	-0.68
Azerbaijan	0.81	0.81	0.73
Bahrain	0.15	0.15	1.08
Barbados	-0.55	-0.55	-0.34
Bolivia	0.48	0.48	1.33
Botswana	-0.25	-0.25	-0.52
Burkina Faso	0.26	0.26	0.31
Burundi	-0.60	-0.60	0.90
Cameroon	0.93	0.93	1.23
Central Africa Republic	-0.65	-0.65	0.56
Chad	-0.35	-0.35	1.1
Comoros	-0.91	-0.91	-2.17
Cote D'Ivoire	1.63	1.63	1.12
Croatia	1.56	1.56	0.67
Dominica	-1.71	-1.71	0.16
Egypt	1.49	1.49	1.24
El Salvador	-0.19	-0.19	0.6
Eritrea	0.15	0.15	-0.05
Estonia	1.07	1.07	-1.51
Ethiopia	0.29	0.29	-1.56
Gabon	0.32	0.32	0.04
Gambia	-0.10	-0.10	-0.33
Ghana	-0.69	-0.69	0.48
Grenada	-0.64	-0.64	0.3
Guatemala	1.56	1.56	1.07
Guinea	0.14	0.14	1.14
Guinea Bissau	-0.13	-0.13	-0.77
Indonesia	0.79	0.79	1.25
Iraq	-0.01	-0.01	0.51
Jordan	-0.18	-0.18	-0.14
Kenya	-0.12	-0.12	-0.68
Kuwait	-0.94	-0.94	0.38
Lebanon	-0.41	-0.41	-0.71
Lesotho	-1.40	-1.40	0.49
Liberia	0.55	0.55	1.09
Madagascar	0.18	0.18	0.66
Malawi	-0.01	-0.01	0.51
Maldives	-0.88	-0.88	-1.72
Mali	0.58	0.58	0.61
Malta	0.93	0.93	0.27
Mauritania	-0.36	-0.36	0.35

Index of Regulation of Pharmaceutical Market by Small and Developing Countries (cont.)

	Regulatory Infrastructure	Private Market Regulation	Public Market Regulation
Mauritius	-0.05	-0.05	0.28
Montenegro	1.09	1.09	-0.79
Morocco	0.55	0.55	0.37
Mozambique	-0.64	-0.64	-0.53
Namibia	1.32	1.32	-0.52
Niger	0.30	0.30	0.60
Nigeria	0.18	0.18	-0.68
Oman	0.63	0.63	-0.72
Pakistan	-0.40	-0.40	0.34
Palestine	-0.55	-0.55	-0.12
Panama	0.60	0.60	-0.29
Peru	0.43	0.43	-0.64
Philippines	0.62	0.62	-0.52
Qatar	0.07	0.07	0.67
Republic of Moldova	0.08	0.08	-0.74
Dominican Republic	0.54	0.54	-0.16
Saint Kitts and Nevis	-1.34	-1.34	-1.79
St. Lucia	-1.39	-1.39	-0.33
St. Vincent and the Grenadines	-0.64	-0.64	0.84
São Tomé and Principe	-0.74	-0.74	-1.88
Saudi Arabia	1.63	1.63	1.26
Senegal	1.18	1.18	-1.06
Seychelles	-2.01	-2.01	0.13
Sierra Leone	0.26	0.26	0.68
Sri Lanka	-0.54	-0.54	-0.38
Suriname	-0.70	-0.70	-0.02
Syrian Arab Republic	-0.36	-0.36	-0.79
Tanzania	0.76	0.76	1.42
Trinidad and Tobago	0.52	0.52	0.63
Tunisia	0.49	0.49	-1.00
Turkey	0.09	0.09	-1.03
Uganda	0.33	0.33	0.58
Uruguay	1.32	1.32	-0.71
Uzbekistan	0.96	0.96	0.07
Yemen	-0.62	-0.62	1.11
Zambia	0.73	0.73	0.39
Zimbabwe	1.63	1.63	0.72

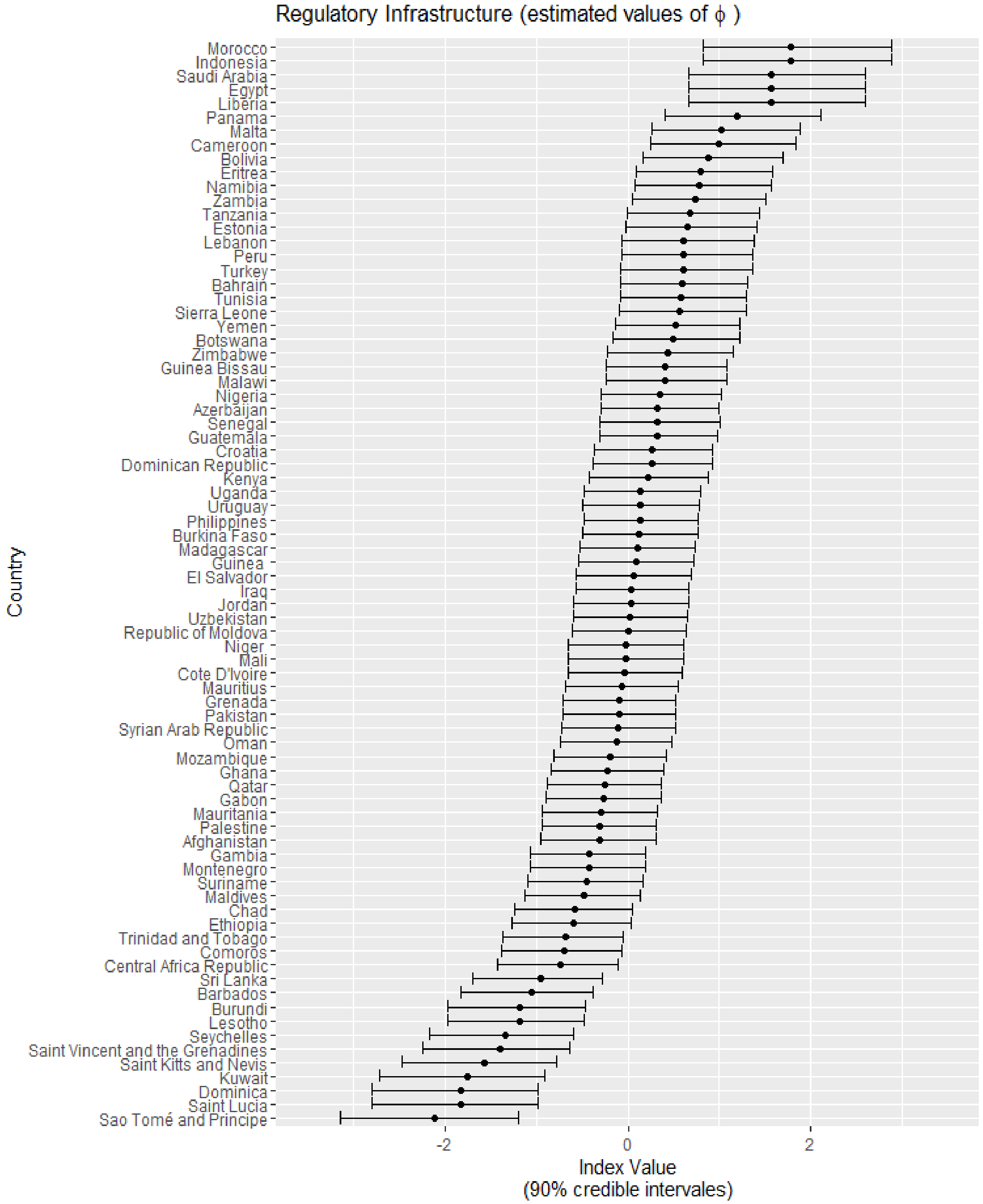

Regulatory Infrastructure (estimated values of ϕ)
Morocco
Indonesia
Saudi Arabia
Egypt
Liberia
Panama
Malta
Cameroon
Bolivia
Eritrea
Namibia
Zambia
Tanzania
Estonia
Lebanon
Peru
Turkey
Bahrain
Tunisia
Sierra Leone
Yemen
Botswana
Zimbabwe
Guinea Bissau
Malawi
Nigeria
Azerbaijan
Senegal
Guatemala
Croatia
Dominican Republic
Kenya
Uganda
Uruguay
Philippines
Burkina Faso
Madagascar
Guinea
El Salvador
Iraq
Jordan
Uzbekistan
Republic of Moldova
Niger
Mali
Cote D'Ivoire
Mauritius
Grenada
Pakistan
Syrian Arab Republic
Oman
Mozambique
Ghana
Qatar
Gabon
Mauritania
Palestine
Afghanistan
Gambia
Montenegro
Suriname
Maldives
Chad
Ethiopia
Trinidad and Tobago
Comoros
Central Africa Republic
Sri Lanka
Barbados
Burundi
Lesotho
Seychelles
Saint Vincent and the Grenadines
Saint Kitts and Nevis
Kuwait
Dominica
Saint Lucia
Sao Tomé and Principe
Country
-2
0
2
Index Value
(90% credible intervales)

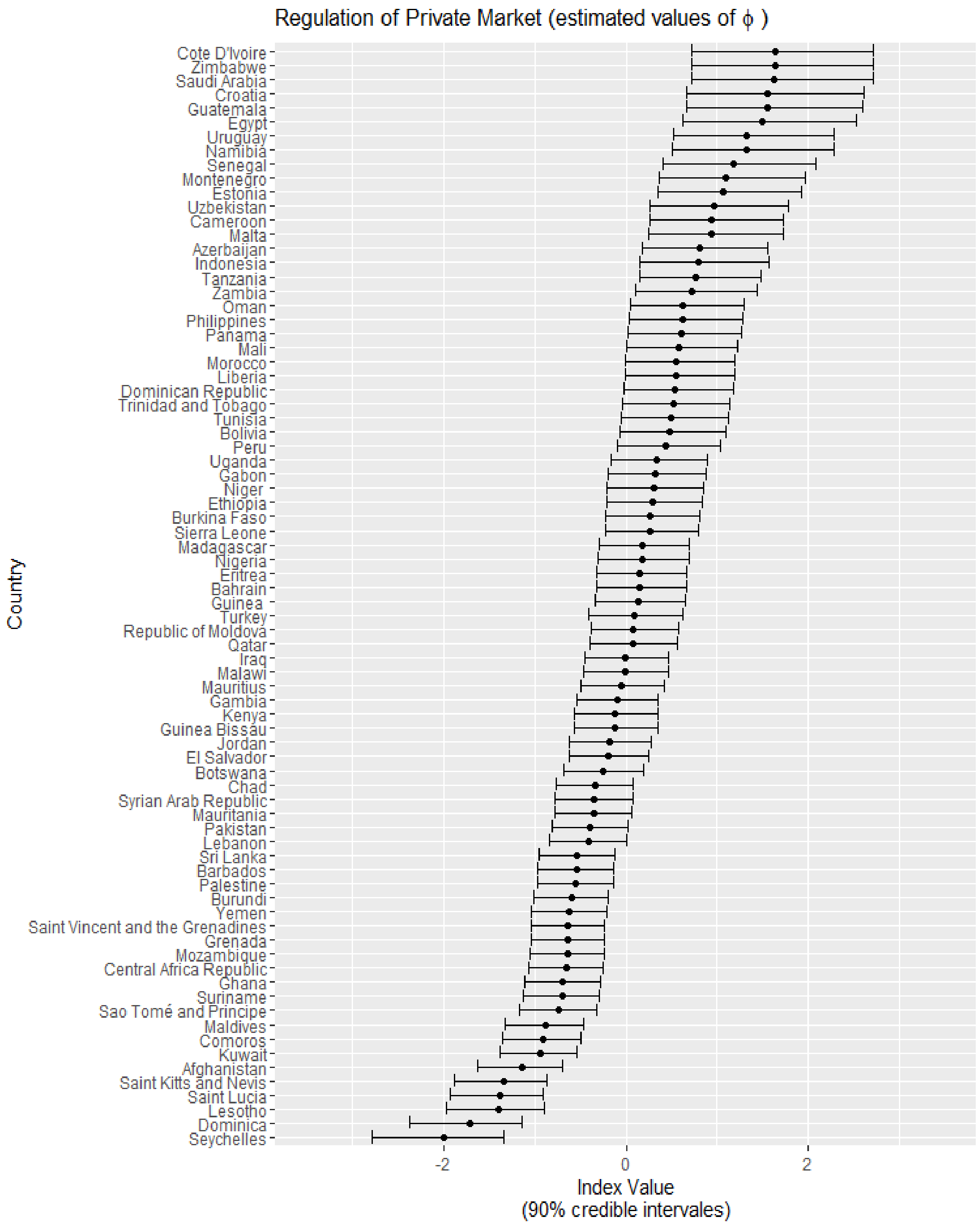

Regulation of Private Market (estimated values of ϕ)
Country
Cote D'Ivoire
Zimbabwe
Saudi Arabia
Croatia
Guatemala
Egypt
Uruguay
Namibia
Senegal
Montenegro
Estonia
Uzbekistan
Cameroon
Malta
Azerbaijan
Indonesia
Tanzania
Zambia
Oman
Philippines
Panama
Mali
Morocco
Liberia
Dominican Republic
Trinidad and Tobago
Tunisia
Bolivia
Peru
Uganda
Gabon
Niger
Ethiopia
Burkina Faso
Sierra Leone
Madagascar
Nigeria
Eritrea
Bahrain
Guinea
Turkey
Republic of Moldova
Qatar
Iraq
Malawi
Mauritius
Gambia
Kenya
Guinea Bissau
Jordan
El Salvador
Botswana
Chad
Syrian Arab Republic
Mauritania
Pakistan
Lebanon
Sri Lanka
Barbados
Palestine
Burundi
Yemen
Saint Vincent and the Grenadines
Grenada
Mozambique
Central Africa Republic
Ghana
Suriname
Sao Tomé and Principe
Maldives
Comoros
Kuwait
Afghanistan
Saint Kitts and Nevis
Saint Lucia
Lesotho
Dominica
Seychelles
-2
0
2
Index Value
(90% credible intervales)

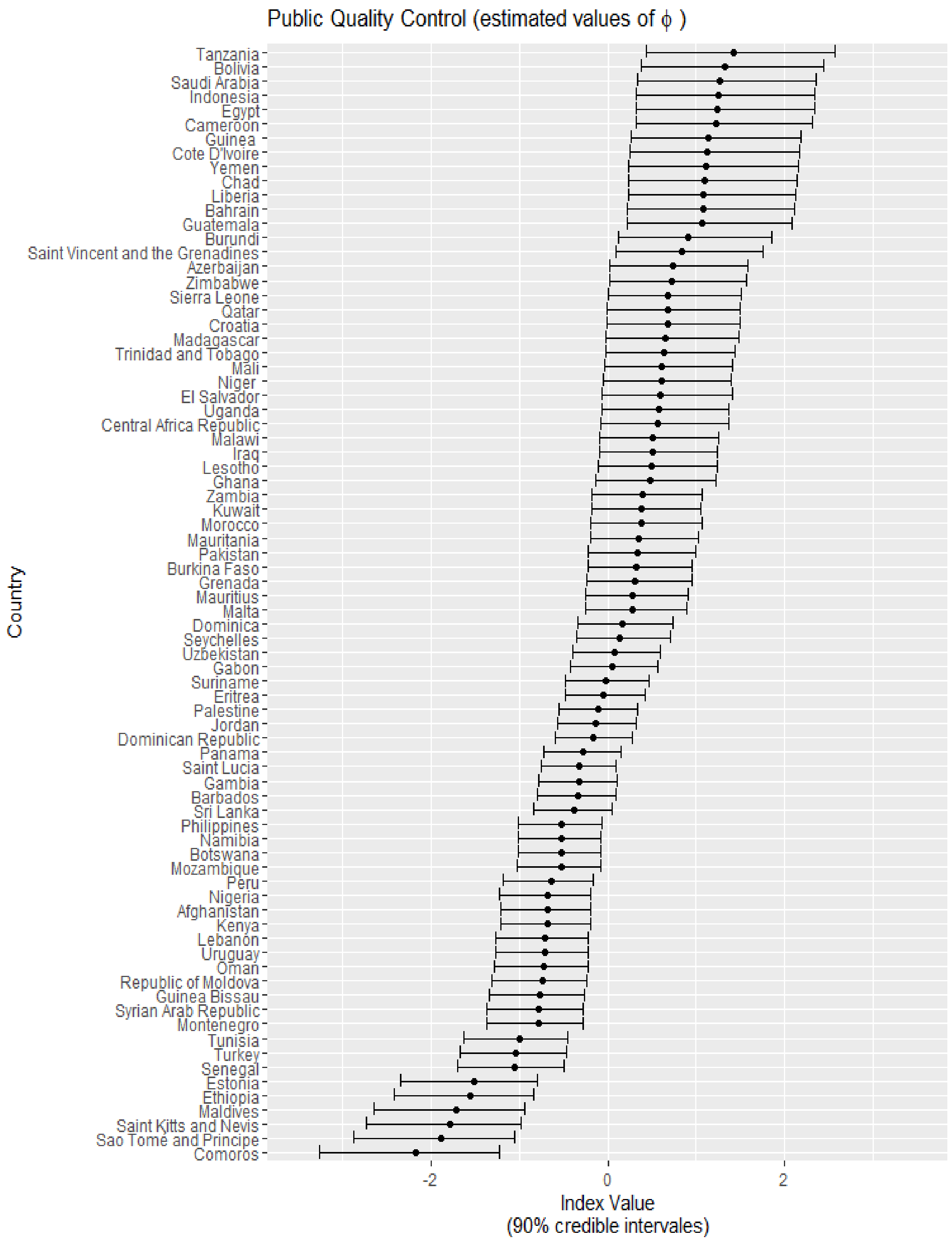

Public Quality Control (estimated values of ϕ)
Country
Tanzania
Bolivia
Saudi Arabia
Indonesia
Egypt
Cameroon
Guinea
Cote D'Ivoire
Yemen
Chad
Liberia
Bahrain
Guatemala
Burundi
Saint Vincent and the Grenadines
Azerbaijan
Zimbabwe
Sierra Leone
Qatar
Croatia
Madagascar
Trinidad and Tobago
Mali
Niger
El Salvador
Uganda
Central Africa Republic
Malawi
Iraq
Lesotho
Ghana
Zambia
Kuwait
Morocco
Mauritania
Pakistan
Burkina Faso
Grenada
Mauritius
Malta
Dominica
Seychelles
Uzbekistan
Gabon
Suriname
Eritrea
Palestine
Jordan
Dominican Republic
Panama
Saint Lucia
Gambia
Barbados
Sri Lanka
Philippines
Namibia
Botswana
Mozambique
Peru
Nigeria
Afghanistan
Kenya
Lebanon
Uruguay
Oman
Republic of Moldova
Guinea Bissau
Syrian Arab Republic
Montenegro
Tunisia
Turkey
Senegal
Estonia
Ethiopia
Maldives
Saint Kitts and Nevis
Sao Tome and Principe
Comoros
-2
0
2
Index Value
(90% credible intervales)

Figure 2. Relation Between Regulatory Infrastructure and the Regulation of Private Market

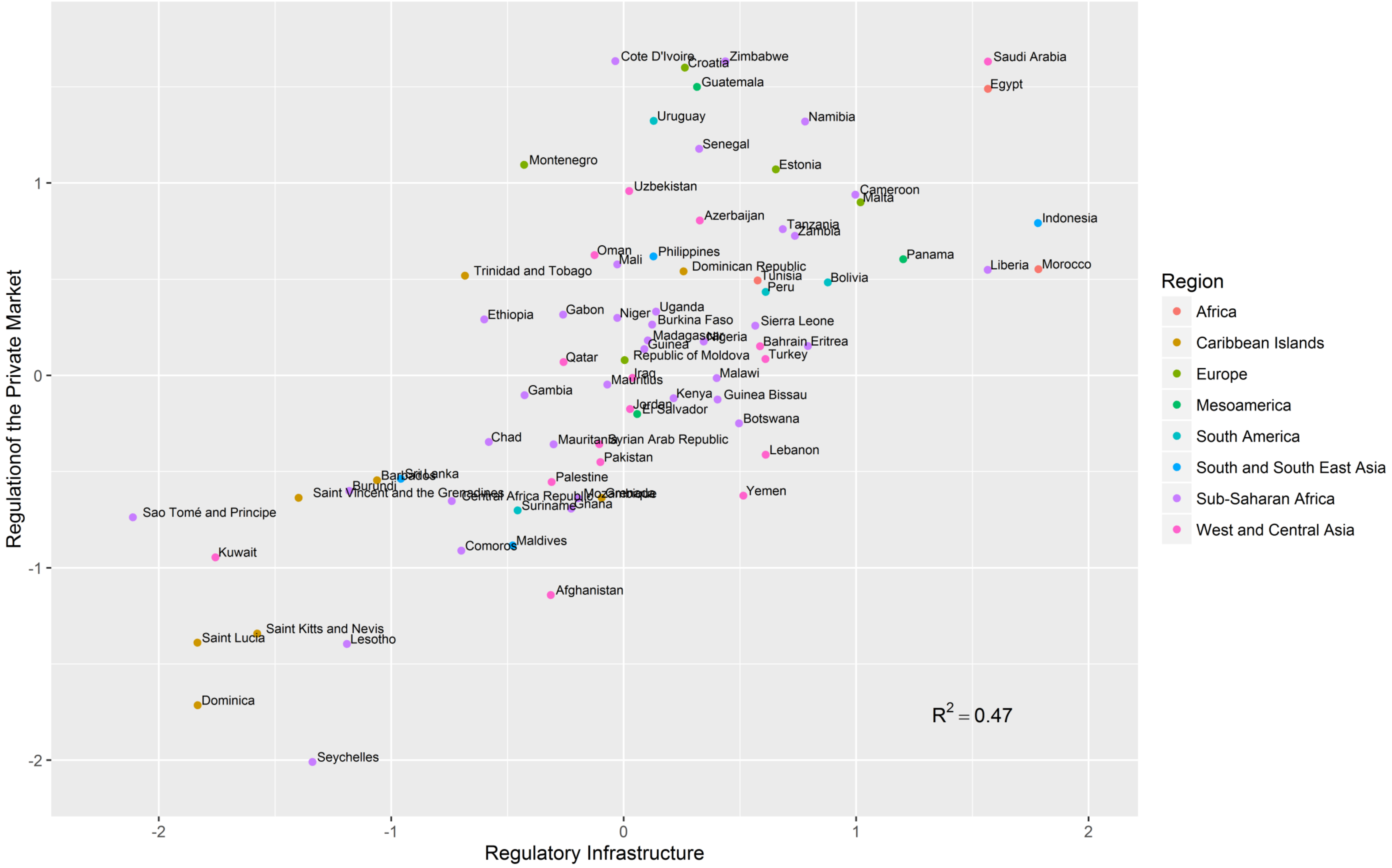

Figure 3. Relation Between Regulatory Infrastructure and the Regulation of the Public Market

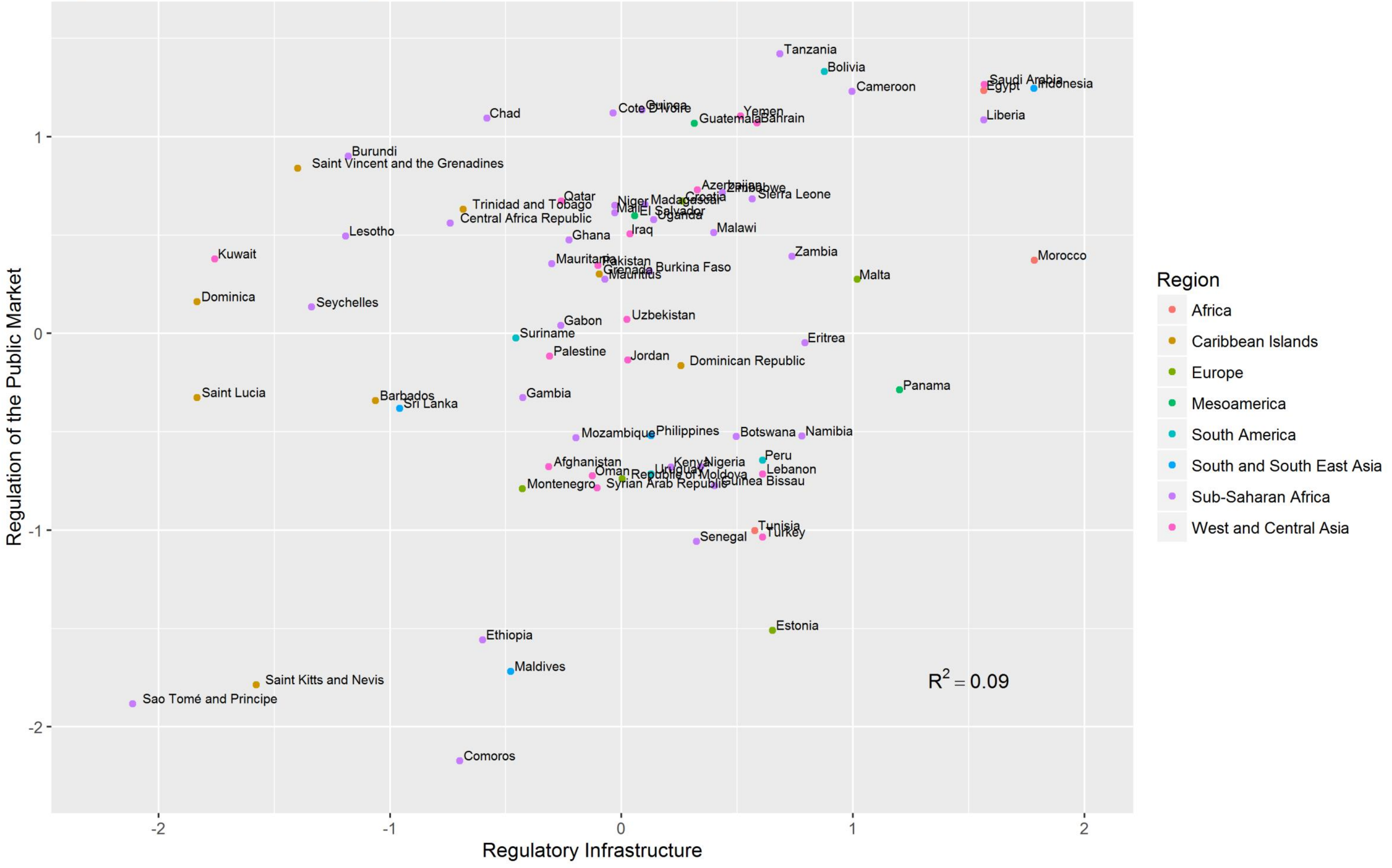

Figure 4. Relation Between Regulation of the Public Market and Regulation of the Private Market

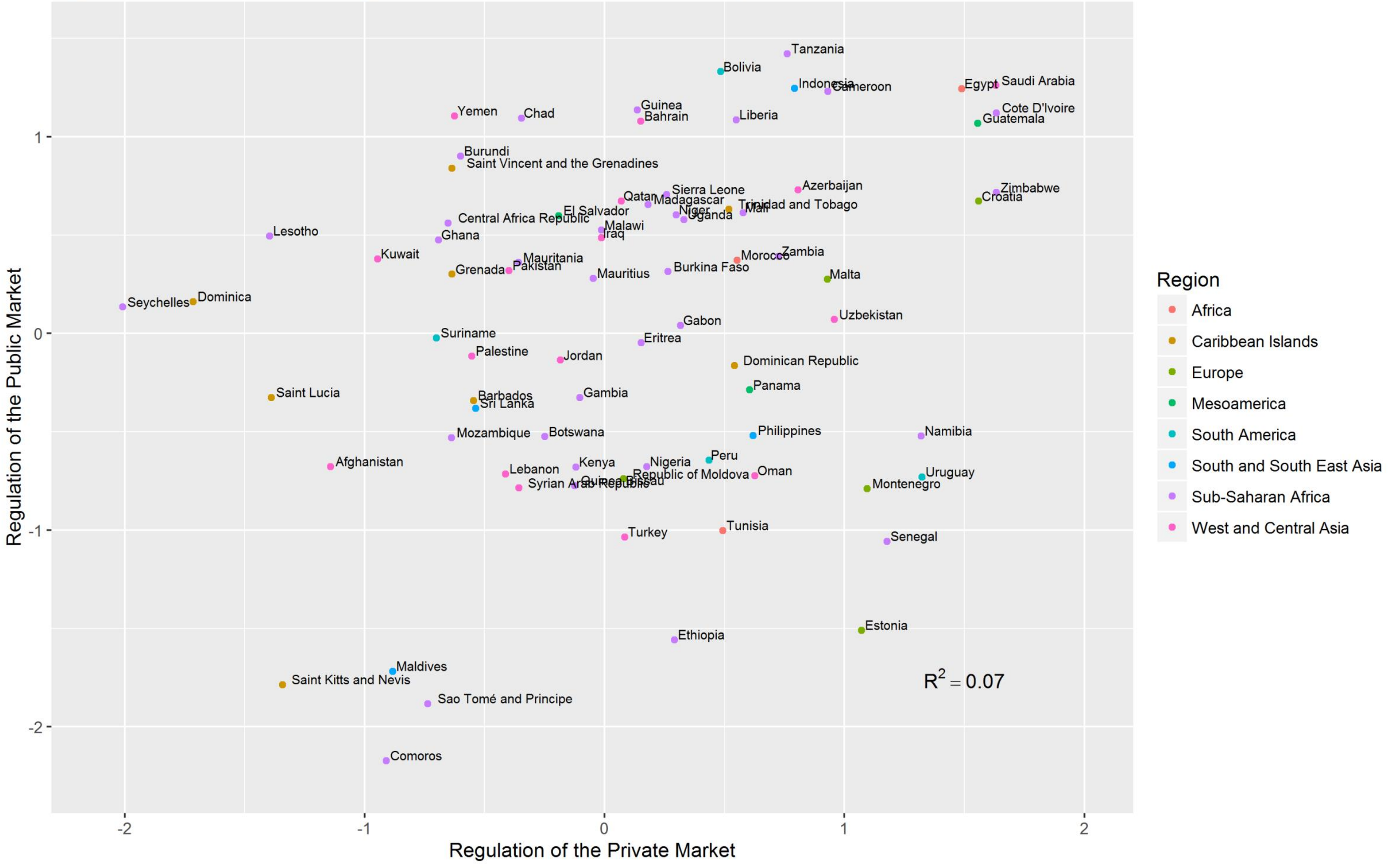

Supplement: Additional file 1: — Country list and additional figures. (PDF 1.04 MB) [file 12992_2016_208_MOESM1_ESM.pdf]
